# Antioxidant and Anticholinesterase Activities of Extracts and Phytochemicals of *Syzygium antisepticum* Leaves

**DOI:** 10.3390/molecules26113295

**Published:** 2021-05-30

**Authors:** Supachoke Mangmool, Issaree Kunpukpong, Worawan Kitphati, Natthinee Anantachoke

**Affiliations:** 1 Department of Pharmacology, Faculty of Science, Mahidol University, Bangkok 10400, Thailand; supachoke.man@mahidol.ac.th; 2 Department of Pharmacognosy, Faculty of Pharmacy, Mahidol University, Bangkok 10400, Thailand; emmi.pornsiri@gmail.com; 3 Department of Physiology, Faculty of Pharmacy, Mahidol University, Bangkok 10400, Thailand; worawan.kit@mahidol.ac.th; 4 Center of Excellence for Innovation in Chemistry, Faculty of Science, Mahidol University, Bangkok 10400, Thailand

**Keywords:** *Syzygium antisepticum*, antioxidant, antioxidant enzyme, anticholinesterase, flavonoids, triterpenoids

## Abstract

Bioassay-guided separation of young leaves extracts of *Syzygium antisepticum* (Blume) Merr. & L.M. Perry led to the isolation of four triterpenoids (betulinic acid, ursolic acid, jacoumaric acid, corosolic acid) and one sterol glucoside (daucosterol) from the ethyl acetate extract, and three polyphenols (gallic acid, myricitrin, and quercitrin) from the methanol (MeOH) extract. The MeOH extract of *S. antisepticum* and some isolated compounds, ursolic acid and gallic acid potentially exhibited acetylcholinesterase activity evaluated by Ellman’s method. The MeOH extract and its isolated compounds, gallic acid, myricitrin, and quercitrin, also strongly elicited DPPH radical scavenging activity. In HEK-293 cells, the MeOH extract possessed cellular antioxidant effects by attenuating hydrogen peroxide (H_2_O_2_)-induced ROS production and increasing catalase, glutathione peroxidase-1 (GPx-1), and glutathione reductase (GRe). Furthermore, myricitrin and quercitrin also suppressed ROS production induced by H_2_O_2_ and induced GPx-1 and catalase production in HEK-293 cells. These results indicated that the young leaves of *S. antisepticum* are the potential sources of antioxidant and anticholinesterase agents. Consequently, *S. antisepticum* leaves are one of indigenous vegetables which advantage to promote the health and prevent diseases related to oxidative stress.

## 1. Introduction

An aging society is a global trend in the 21st century. Many countries are increasing in their aging population. The advances in medical knowledge and technology together with the improvements in sanitation and education of people result in a decrease in early- and mid-life mortality [1]. Therefore, the challenge now lies in continuous decline in late-life mortality and increasing longevity. Physiological function impairments and homeostatic imbalance naturally take place in the period of old age and cause an increased risk of aging-associated diseases such as neurodegenerative diseases, cardiovascular diseases, atherosclerosis, cancer, and metabolic disorders [2,3]. One of the important causes of aging and many chronic diseases is oxidative stress, an imbalance between reactive oxygen and nitrogen species (RONS) accumulation and anti-oxidation defense in cells and tissues [2,3]. Intrinsic RONS are generated by various physiologic and pathophysiologic processes, and exogenous sources of RONS are mainly produced from pollution, radiation, and some chemicals [2]. The excess of cellular RONS levels can induce carbohydrates, proteins, lipids, DNA, cells, and tissue damages [2,4]. In addition to the biological aging process, various factors are associated with functional decline such as genetic variation, lifestyle, nutritional, and living environment [3].

Alzheimer’s disease (AD) is a neurodegenerative disease causing progressive cognitive and memory loss, behavior changes, daily activity impairment, and various neuropsychiatric symptoms [5]. The pathophysiology of AD has been described in many hypotheses related to amyloid beta (β-amyloid) aggregation, tau protein hyperphosphorylation, oxidative stress, mitochondria dysfunction, cholinergic dysfunction, and metal dyshomeostasis [6,7]. Up to now, AD cannot be cured and there are only a few drugs used for symptomatic treatments in order to delay the severity of AD [6]. Acetylcholinesterase inhibitors (AChEIs), including donepezil, rivastigmine, and galantamine play an important role in treatment of mild to severe dementia [6]. Moreover, memantine, an N-methyl-D-aspartate (NMDA) receptor antagonist, has been used for treatment of moderate to severe AD and other neurodegenerative diseases via inhibition of glutamate-mediated neurotoxicity [8]. In addition to the pharmacotherapeutics, many non-pharmacological treatments have been used for improving the quality of life, cognitive function, mental, and physical health. Many edible plants play an important role in the prevention and delay of aging and neurodegenerative diseases [6]. *Bacopa monnieri* (L.) Wettst. [9], *Centella asiatica* (L.) Urb. [10], and *Curcuma longa* L. [11] are considered to have beneficial health effects, especially for memory and cognitive function. They have attracted considerable attention for AD treatment because of their multi-target pharmacological mechanisms, such as antioxidant, anti-inflammation, anticholinesterase, β-amyloid aggregation inhibition, and neuroprotective properties [9,10,11]. Consequently, this study investigated for antioxidant, acetylcholinesterase inhibition activities, and bioactive compounds from *Syzygium antisepticum* (Blume) Merr. & L.M. Perry, synonym *S. gratum* (Wight) S.N. Mitra. *S. antisepticum* is an evergreen tree in the family Myrtaceae and widely distributed in Southeast Asia [12]. Its young leaves are reddish and consumed as a local vegetable in Thailand with a slightly astringent and sour taste [12,13]. The leaves of *S. antisepticum* are high in phenolic and flavonoid contents that possess antioxidant and free radical scavenging properties. [14,15]. The biological studies of *S. antisepticum* leaves have attracted growing interest because of the significant role in inhibiting oxidative stress. It has been demonstrated that the *S. antisepticum* leaf extracts possess free radical scavenging and antioxidant activities evaluated in vitro by the 1,1-diphenyl-2-picrylhydrazyl (DPPH) and ABTS free radical-scavenging activities, ferric reducing antioxidant power (FRAP) and ferrous ion-chelating (FIC) assays [13,15,16], and the intracellular antioxidant activity due to the inhibition of nitric oxide (NO) production in rat peritoneal macrophages RAW 264.7 cells [13]. The leaf aqueous extract could induce cytoprotective enzyme, the heme oxygenase-1 (HO-1) enzymatic activity in C57BL/6J mice [12]. Moreover, the administration of leaf aqueous extract at dose of 0.1 g/kg body weigh/day for 7 days in blood transfusion-dependent thalassemia patients could enhances plasma antioxidant capacity evaluated by FRAP assay [17]. The plant extracts have ability in vascular protective effect by improving blood reduced glutathione and decreasing in plasma malondialdehyde, NO metabolites, and blood superoxide anion formations in phenylhydrazine induced rats [13].

Apart from *S. antisepticum*, various plants in the genus *Syzygium* have been reported on their pharmacological activities, including antioxidant, anticancer, anti-inflammation, antimicrobial, antidiabetic [18], and hepatoprotective [19] activities. Moreover, some plants in this genus have been revealed to contain a variety of phytochemical constituents such as flavonoids from *S. aqueum* (Burm.f.) Alston, *S. aromaticum* (L.) Merr. & L.M.Perry, *S. cumini* (L.) Skeels, *S. guineense* (Willd.) DC. [18], *S. samarangense* (Blume) Merr. & L.M.Perry [18,20], *S. jambos* (L.) Alston [19], and *S. malaccense* (L.) Merr. & L.M.Perry [21]; phenols from *S. aqueum*, *S. aromaticum*, *S. samarangense* [18], *S. jambos* [19], and *S. leucoxylon* Korth. [22]; chromones from *S. aromaticum*; terpenoids from *S. aromaticum*, *S. cumini*, *S. guineense*, and *S. samarangense*; sterols from *S. aromaticum* and *S. cumini* [18].

Although antioxidant properties both in vitro and in vivo of *S. antisepticum* leaf extracts have been demonstrated, various antioxidant mechanisms in the cellular system, anticholinesterase properties, and active constituents of the *S. antisepticum* young leaves have not been fully understood. Therefore, this study aimed to evaluate the effects of *S. antisepticum* young leaf extracts on anticholinesterase and antioxidant activities and investigate their active phytochemical constituents. In addition, we also investigated the effects of the MeOH extract and its chemical constituents on inhibition of cellular oxidative stress induced by H_2_O_2_ and on the synthesis of antioxidant enzymes determined by measurement of mRNA levels in HEK-293 cells.

## 2. Results and Discussion

### 2.1. Bioassay-Guided Fractionation

The young leaves of *S. antisepticum* extracts were screened for antioxidant activity by using DPPH method and acetylcholinesterase inhibitory activity using Ellman’s method by microplate spectroscopic method. The results showed that the ethyl acetate and methanol extracts exhibited DPPH radical scavenging activity with more than 50% inhibition at concentration of 50 µg/mL. The antioxidant activity of the extracts are corresponding to several previous studies which have demonstrated the antioxidant properties of *S. antisepticum* leaf extracts investigated by using DPPH, FRAP, and ABTS assays [13,15,16]. Moreover, it was found the first time that the methanol extract could inhibit acetylcholinesterase enzyme activity by 61.90 ± 0.08% at concentration of 300 µg/mL (Table 1). Therefore, bioactive compounds of the ethyl acetate and methanol extracts were selected for further investigation.

**Figure 1:**
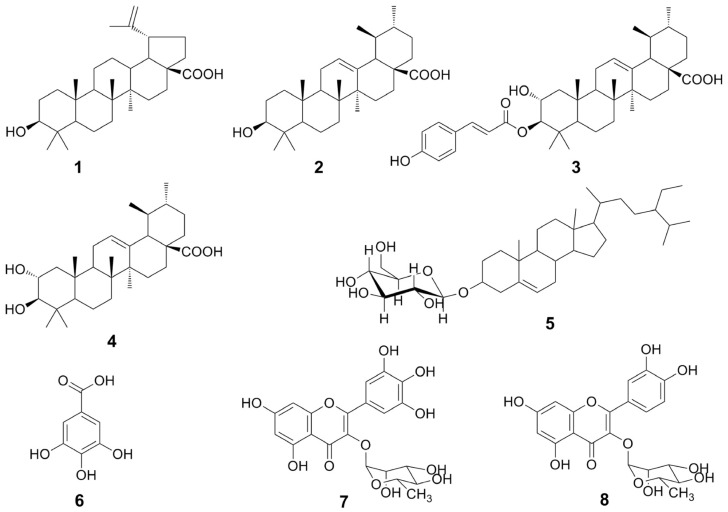
Isolated compounds from *S. antisepticum* young leaves.

Antioxidant and anticholinesterase activities-guided fractionation via TLC bioautographic assay of the ethyl acetate and methanol extracts of *S. antisepticum* young leaves resulted in the isolation of five triterpenoids **1**–**5**, one polyphenolic acid **6**, and two flavonoids **7**–**8**. The structures of the isolated compounds were identified to be betulinic acid (**1**) [23,24,25], ursolic acid (**2**) [26], jacoumaric acid (**3**) [27], corosolic acid or 2α-hydroxyursolic acid (**4**) [26], daucosterol or stigma-5-en-3-*O*-*β*-glucoside (**5**) [28], gallic acid (**6**) [29], myricitrin or myricetin-3-*O*-α-L-rhamnoside (**7**), and quercitrin or quercetin 3-*O*-α-L-rhamnoside (**8**) [30] (Figure 1) based on their IR, UV, NMR, and MS spectroscopic data (Supplementary material) and by comparison with previously reported spectroscopic and physical data. Although it has been reported that *S. antisepticum* leaves contain high phenolic and flavonoid contents [14,15], the specific phytochemicals have not been revealed. Therefore, the obtained results that *S. antisepticum* leaves contain various types of chemical constituents, including triterpenoids **1**–**4**, sterol **5**, polyphenolic acid **6**, and flavonoid glycosides **7**–**8**, are reported for the first. These results are consistent with the phytochemicals that reported from other plants in the same genus [18]. Interestingly, we found that myricitrin (**7**) is a predominant flavonoid in the *S. antisepticum* leaves, the same as results revealed from the leaves of the other *Syzygium* plants, *S. samarangense* [20] and *S. malaccense* [21] although quercetin glycosides more commonly present in plants than myricetin glycosides [31].

The isolated compounds **1**–**8** were evaluated for acetylcholinesterase inhibitory and DPPH scavenging activities. The results showed that among those of compounds isolated from *S. antisepticum* leaves, ursolic acid (**2**) and gallic acid (**6**) exhibited anticholinesterase activity with more than inhibitory effect of 50% at a concentration of 100 μg/mL and had the IC_50_ values of 159.32 ± 1.34 μM and 161.06 ± 9.52 µM, respectively (Table 1). This result is related to the previous studies representing the action of ursolic acid (**2**) [32,33] and gallic acid (**6**) [34] as potent AChEIs. Moreover, it has been reported that ursolic acid (**2**) exhibits multi-target properties for ameliorating disorders associated with CNS through inhibitory activities against oxidative stress and inflammation [35,36,37]. Moreover, the neuroregeneration effect of ursolic acid (**2**) resulted in the improvement of cognitive functions of *β*-amyloid-induced neurotoxicity and memory impairments in mice [35]. Many studies reported that gallic acid (**6**) has been shown to have several pharmacological activities related to the management of AD and other neurological diseases, including neuroprotective and anti-inflammatory effects [7]. Apart from the inhibitory effect against AChE activity, gallic acid (**6**) could also attenuate the activity of *β*-secretase (BACE-1), an enzyme in the biogenesis process of *β*-amyloid or amyloidogenic pathway [38,39]. It has been reported that gallic acid (**6**) could inhibit *β*-amyloid aggregation and *β*-amyloid plaques formation both in vitro and vivo assays [40,41]. Therefore, ursolic acid (**2**) and gallic acid (**6**), natural substances found in plants, are promising multifunctional agents for protection or treatment of neurodegenerative disorders.

Furthermore, it was found that the polyphenolic compounds, gallic acid (**6**), myricitrin (**7**), and quercitrin (**8**) showed considerable DPPH scavenging activity with the IC_50_ values of 14.46 ± 0.29, 31.01 ± 0.90, and 38.34 ± 0.74 μM, respectively (Table 1). Therefore, the polyphenolic compounds **6**–**8** corresponded to the antioxidant effect of the *S. antisepticum* young leaves extracts. The results related to various reports that phenolic acids and flavonoids are important antioxidants in plants. Gallic acid (**6**) was found to be a strongest DPPH free radical scavenging ability in the extract of *S. antisepticum* leaves and plays an important natural antioxidant in many plants [42]. It has been reported that both myricitrin (**7**) and quercitrin (**8**) exhibited antioxidant effects as determined by free radical scavenging and FRAP assays [20,21,43,44,45]. It was well known that phenolic compounds as well as flavonoids are bioactive chemical constituents found in many plants. These compounds possess antioxidant properties because they are able to scavenge reactive oxygen and nitrogen species. Several previous studies have been reported that phenolic compounds positively correlated with antioxidant activity [42,46,47,48].

### 2.2. Effects of Methanol Extract and Flavonoid Glycosides from S. antisepticum on Cell Viability of HEK-293 Cells

**Figure 2:**
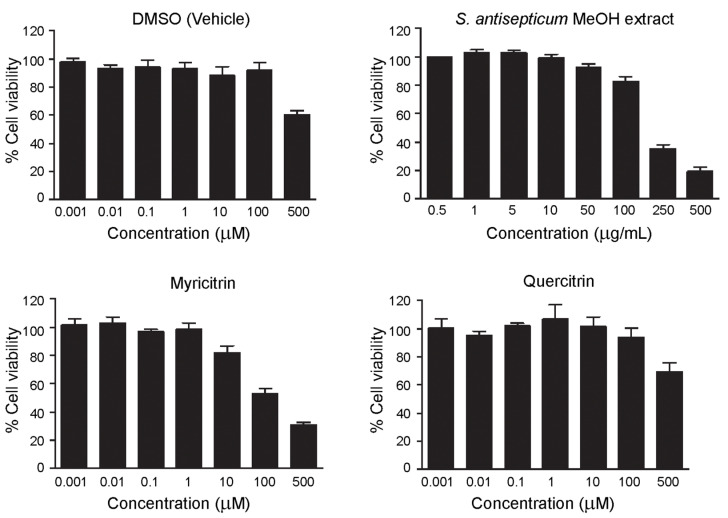
Cytotoxicity profiles of the extract from *S. antisepticum* and flavonoid glycosides **7** and **8**; HEK-293 cells were treated with methanol (MeOH) extract (0.5–500 μg/mL), myricitrin (**7**) (0.001–500 μM), or quercitrin (**8**) (0.001–500 μM) for 36 h. Data are shown as the percentage of cell viability (mean ± SEM) (n = 4).

The results from bioassay-guided separation led to the further studies of cellular antioxidant effects and mechanism of actions of the methanol extract of *S. antisepticum* young leaves, and its isolated compounds in HEK-293 cells. The potent DPPH radical scavenging activities among isolated compounds from *S. antisepticum* were observed in gallic acid (**6**), myricitrin (**7**), and quercitrin (**8**) (Table 1). In this study, the flavonoid glycoside **7** and **8** were selected to determine antioxidant activities in HEK-293 cells. The methanol extract and flavonoid glycosides **7** and **8** were first investigated the cell cytotoxicity using MTT assay for determining the optimal concentrations for assessment of cellular antioxidant activity. As shown in Figure 2, the methanol extract at concentrations of 0.5–100 μg/mL were not toxic to the cells. Treatment of HEK-293 cells with myricitrin (**7**) with concentrations more than 10 μM or quercitrin (**8**) more than 100 μM exhibited cytotoxic effects which numbers of cell viabilites were less than 80%. In order to exclude the toxic effects and allow approximately 80–90% cell survival, the concentrations used in further experiments were not more than 100 µg/mL for the methanol extract, and not more than 10 µM for the flavonoid glycosides **7** and **8**.

### 2.3. Effects of Methanol Extract and Flavonoid Glycosides from S. antisepticum on H_2_O_2_-Induced Intracellular ROS Production and mRNA Expression of Antioxidant Enzymes in HEK-293 Cells

Antioxidants are the substances that have potential activities for retarding or preventing the oxidation reaction and scavenging of ROS (e.g., superoxide and hydroxyl radical). Oxidative stress causes ROS overproduction inducing cell injury and tissue damage which contribute to the pathogenesis of chronic diseases [2,4]. Several antioxidant enzymes, including catalase, glutathione peroxidase-1 (GPx-1), glutathione reductase (GRe), superoxide dismutase (SOD), and heme oxygenase-1 (HO-1) and non-enzymatic antioxidant compounds such as phenolic compounds and flavonoids play an important role on antioxidant defense mechanisms (e.g., inhibition of ROS production, repair of oxidative damages, and scavenging of free radicals) [4]. For instance, superoxide anion is dismutated by SODs to generate oxygen molecule and H_2_O_2_. After that, elimination of H_2_O_2_ is efficiently eliminated by GPx and catalase [49]. Thus, assessments of these antioxidant enzymatic activities and their synthesis might be used as the sensitive biomarkers in responses to oxidative stress and oxidative damage in cells and tissues.

**Figure 3:**
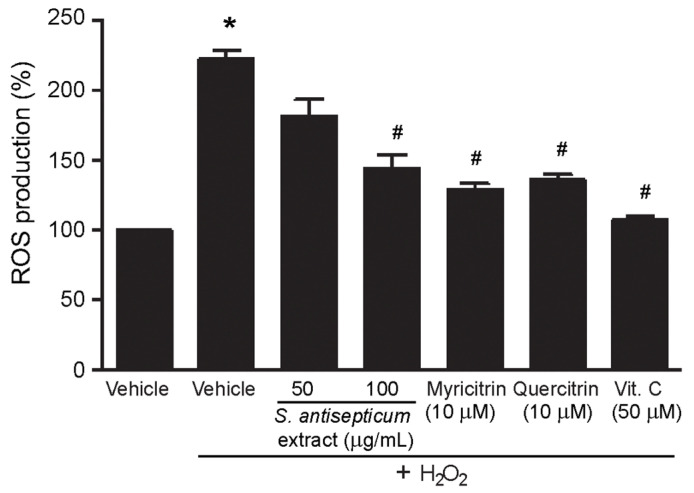
Cellular antioxidant effects of the methanol (MeOH) extract and flavonoid glycosides **7** and **8** from *S. antisepticum*; HEK-293 cells were treated with either methanol extract (50 and 100 μg/mL), myricitrin (**7**) (10 μM), quercitrin (**8**) (10 μM), or vitamin C (10 μM) for 3 h and then treated with H_2_O_2_ (100 μM) for 1 h. Data are shown as the percentage of ROS levels (mean ± SEM) as compared with that in control (vehicle; DMSO) group (n = 4). * *p* < 0.05 versus control; ^#^ *p* < 0.05 versus the H_2_O_2_ group.

Even though intracellular antioxidant activity due to the inhibition of nitric oxide (NO) production in rat peritoneal macrophages RAW 264.7 cells of *S. antisepticum* leaf extracts have been reported [13], the intracellular antioxidant effects of the leaf extract of *S. antisepticum* on suppression of ROS production and the upregulation of some antioxidant enzymes in the cells have not been determined. Therefore, antioxidant effects of the methanol extract of *S. antisepticum* young leaves and flavonoid glycosides, myricitrin (**7**), and quercitrin (**8**), on inhibition of H_2_O_2_-induced intracellular ROS production were determined by using a fluorescent probe DCFH-DA. As shown in Figure 3, incubation the cells with H_2_O_2_ robustly increased the intracellular ROS levels compared to that of vehicle (DMSO) group. Treatment with the methanol extract significantly attenuated H_2_O_2_-induced ROS production in a dose-dependent way. In addition, treatment with either 10 μM of myricitrin (**7**) or quercitrin (**8**) significantly reduced ROS production and had effects similar to vitamin C (ascorbic acid; potent antioxidant) (Figure 3). Collectively, these results demonstrated that both myricitrin (**7**) and quercitrin (**8**), the active constituents found in young leaves of *S. antisepticum*, possess the antioxidant effects by reducing intracellular ROS production.

**Figure 4:**
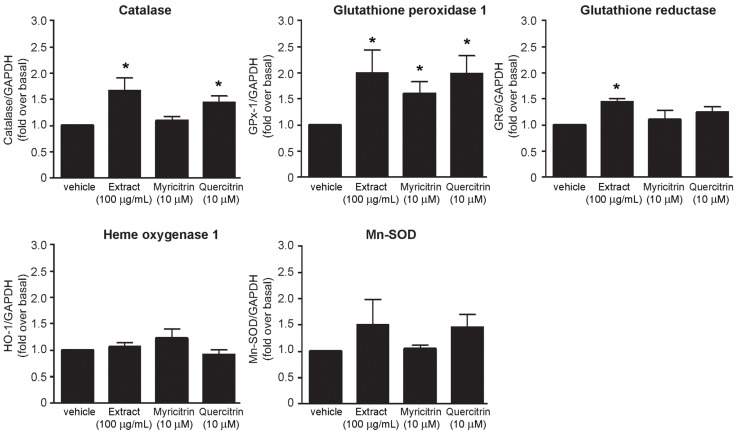
Effects of the methanol extract and flavonoid glycosides **7** and **8** from *S. antisepticum* on upregulation of antioxidant mRNA levels; HEK-293 cells were treated with either methanol extract of *S. antisepticum* (100 μg/mL), myricitrin (**7**) (10 μM), or quercitrin (**8**) (10 μM) for 6 h. The relative mRNA expression levels were quantified and shown as mean ± SEM as fold over basal (vehicle, DMSO) (n = 4). * *p* < 0.05 versus control.

Upregulation of cellular antioxidant enzymes can be determined by measurements of mRNA and protein levels, including enzymatic activities in the cells. Several antioxidant enzymes (e.g., catalase, GPx-1, GRe, HO-1, SOD) are the main components of endogenous defense mechanism on inhibition of oxidative stress in many cells and tissues to protect oxidative damage of the cells. Due to treatment with the methanol extract and flavonoid glycosides **7** and **8** from *S. antisepticum* were able to inhibit H_2_O_2_-induced ROS production (Figure 3), upregulation antioxidant enzymes synthesis by measurement of mRNA levels was further investigate. As shown in Figure 4, treatment with the methanol extract of *S. antisepticum* young leaves significantly increased mRNA levels of catalase, GPx-1, and GRe, and tended to increase Mn-SOD mRNA level in HEK-293 cells. In addition, treatment with myricitrin (**7**) significantly induced GPx-1 mRNA expression while treatment with quercitrin (**8**) significantly increased mRNA levels of catalase and GPx-1 (Figure 4). However, mRNA expressions of GRe, HO-1, and Mn-SOD did not change after treatment with the flavonoid glycosides. Collectively, these data demonstrated that methanol extract of *S. antisepticum* young leaves and its chemical constituents, myricitrin (**7**) and quercitrin (**8**), inhibited oxidative stress by upregulation of antioxidant enzyme synthesis. Consequently, the methanol extract and isolated flavonoid glycosides **7** and **8** from *S. antisepticum* young leaves could attenuate oxidative stress by increasing mRNA expression of catalase, GPx-1, and GRe in HEK-293 cells. The results related to the previous studies on the antioxidation effects of the *S. antisepticum* leaves as mentioned earlier.

The cellular antioxidant effects of the isolated flavonoid glycosides **7** and **8** are related to many previous reports. Myricitrin (**7**) and its derivatives were also discovered to be active compounds in *S. malaccense* leaf extract against high glucose (30 mM)-induced oxidative stress in ARPE-19 (retinal pigment epithelium) due to the decrease in intracellular ROS generation and upregulation of various antioxidant enzyme genes, such as catalase, GPx-1, GPx-2, and Mn-SOD [50]. It could also inhibit ROS generation and glutathione (GSH) depletion in stressed human keratinocytes (HaCaT cells) induced by sodium arsenite [20].

Moreover, quercitrin (**8**) is reported to have a cytoprotective effect against oxidative stress-mediated cell damage and apoptosis by the exposure of Chinese hamster lung fibroblast (V79-4) and human skin keratinocyte (HaCaT) cells to H_2_O_2_ [45] and ultraviolet (UV) B radiation [51], respectively. The effects were determined by the reduction of intracellular ROS generation [45,51] and lipid peroxidation [45] in the cells treated with quercitrin. However, quercetin glycosides, quercitrin and rutin, showed no effect on HO-1 protein and mRNA expression and H_2_O_2_-induced intracellular ROS production and cytotoxicity in mouse macrophage (RAW264.7) cells which could be inhibited by treatment the cells with quercetin [52].

## 3. Materials and Methods

### 3.1. General

Electrothermal IA 9100 digital melting point apparatus (Electrothermal, Staffordshire, UK) and Jasco DIP-370 digital polarimeter (Jasco, Easton, CA, USA), 50 mm microcell were used for measuring melting points (m.p., °C) and optical rotations of the isolated compounds, respectively. Shimadzu UV-2600 UV-Vis (Shimadzu, Kyoto, Japan) and Nicolet 6700 FT-IR (Thermo Fisher Scientific, Waltham, MA, USA) spectrometers were used for recording UV and IR spectra, respectively. ^1^H-NMR, ^13^C-NMR, and 2D NMR spectra were acquired using Bruker AV 500 spectrometer (Bruker, Rheinstetten, Germany). Dimethyl sulfoxide-*d*_6_, methanol-*d*_4_, and pyridine-*d*_5_ (Merck, Darmstadt, Germany) were used as NMR solvents. EI-MS data were acquired using a Thermo Finnigan Polaris Q (Thermo Fisher Scientific, Waltham, MA, USA).

Thin layer chromatography (TLC) was carried out on silica gel 60 GF254 (layer thickness 0.2 mm, Merck, Darmstadt, Germany). Column chromatography (CC) was performed by using silica gel 60 (63–200 µm, Merck, Darmstadt, Germany), silica gel P60 (40–63 µm, Silicycle, QC, Canada), or reverse phase silica gel C-18 (40–63 µm, Silicycle, Quebec, Canada) as a stationary phase.

### 3.2. Chemicals

Acetylthiocholine iodide (ATCI), galantamine, acetylcholinesterase type VI-S lyophilized powder, 5,5′-dithiobis-(2-nitrobenzoic acid) (DTNB), ascorbic acid, and 2,2-diphenyl-1-picrylhydrazyl radical (DPPH), 6-hydroxy-2,5,7,8-tetramethylchroman- 2-carboxylic acid (Trolox^®^), 2′,7′-dichlorodihydrofluorescein diacetate (DCFH-DA), 3-[4,5-dimethylthiazol-2-yl]-2,5-diphenyl tetrazolium bromide (MTT), bovine serum albumin (BSA), were purchased from Sigma-Aldrich (St. Louis, MO, USA). Hydrogen peroxide and dimethyl sulfoxide (DMSO) were purchased from Merck (Darmstadt, Germany). Cell culture products (DMEM, FBS, penicillin/streptomycin solution, and 0.25% trypsin-EDTA) were obtained from Gibco (Grand Island, NY, USA). The other chemicals were commercially available and analytical grade.

### 3.3. Plant Materials

The young leaves of *S. antisepticum* were collected from Ubonratchathani Province, Thailand. The voucher specimen (BKF number 183766) of *S. antisepticum* was identified by Mr. Sukhid Ruengruea (botanist of Office of the Forest Herbarium, Thailand) and has been kept at the Forest Herbarium, Department of National Parks, Wildlife and Plant Conservation, Bangkok, Thailand.

### 3.4. Extraction and Isolation

The young leaves of *S. antisepticum* were dried in a hot air oven at 50 °C for 48 h. and grounded to coarse powder by a cutting mill. The ground dried leaves (613.2 g) were successively extracted by maceration at room temperature with hexane (2 L × 4 times), ethyl acetate (2 L × 6 times), and methanol (2 L × 5 times), respectively. The extracts were filtered through Whatman No.1 filter paper and the solvents were removed under reduced pressure using a rotary evaporator (Büchi, Flawil, Switzerland), followed with a freeze dryer (Labconco, Kansas, MO, USA) to give dried hexane extract (9.3 g), EtOAc extract (18.2 g), and MeOH extract (74.0 g). The crude extracts were stored at −20 °C until analysis.

The EtOAc extract (17.0 g) was coarsely separated by a short column chromatography (CC) on silica gel and eluted with Me_2_CO–hexane and MeOH–Me_2_CO gradient solvent systems to produce six fractions (A1–A6). Fraction A4 (8.9 g) was rechromatographed using silica gel CC with gradient elution of Me_2_CO–hexane and MeOH–Me_2_CO to yield seven fractions (B1–B7). Fraction B4 (1.2 g) was separated with silica gel CC using Me_2_CO–hexane and MeOH–Me_2_CO as gradient eluents to obtain six fractions (C1–C6). Fraction C3 was purified by recrystallization from MeOH/CH_2_Cl_2_ to afford compound **1** (betulinic acid) (8.8 mg) as white needles. The mother liquor (562.3 mg) from fraction C3 was further subjected to silica gel CC and eluted with Me_2_CO–hexane and MeOH–Me_2_CO gradient solvent systems to provide seven fractions (D1–D7). Fraction D3 (137.7 mg) was rechromatographed on silica gel CC using a gradient elution with Me_2_CO–hexane and MeOH to yield sevent fractions (E1–E7). Fraction E3 was further isolated by recrystallization from MeOH/CH_2_Cl_2_ to give compound **2** (ursolic acid) (74.1 mg) as a white powder. Fractions B6 (2.8 g) was separated over silica gel CC employing an elution gradient of MeOH–CH_2_Cl_2_ to produce eight fractions (F1–F8). Fraction F3 (1.7 g) was rechromatographed using silica gel CC with a gradient elution of MeOH–CH_2_Cl_2_ to afford seven fractions (G1–G7). The recrystallization of fraction G5 from MeOH/CH_2_Cl_2_ provided compound **3** (jacoumaric acid) (168.6 mg) as a white powder. Fraction A5 (2.4 g) was separated by silica gel CC using an elution gradient of MeOH–CH_2_Cl_2_ to yield seven fractions (H1–H7). Fraction H3 (329.6 mg) was rechromatographed over silica gel CC using gradient solvent system containing MeOH-CH_2_Cl_2_ and further purified by recrystallization from MeOH/CH_2_Cl_2_ to obtain compound **4** (corosolic acid, 2α-hydroxyursolic acid) (43.5 mg) as a white powder. Fraction H5 (483.4 mg) was subjected to silica gel CC and eluted with MeOH–CH_2_Cl_2_ gradient solvent system to afford five fractions (I1–I5). Compound **5** (daucosterol, stigma-5-en-3-*O*-*β*-glucoside) (50.0 mg) was obtained as a white powder by recrystallization of fraction I4 from MeOH/CH_2_Cl_2_. (See more details of CC in supplementary material)

The MeOH extract (64.6 g) was separated by short CC over silica gel using MeOH–CH_2_Cl_2_ as a gradient eluent to produce five fractions (A1–A5). Fraction A4 (18.4 g) was further fractionated using silica gel CC and eluted with MeOH–CH_2_Cl_2_ gradient solvent system to yield six fractions (B1–B6). Fraction B5 (3.6 g) was further separated over silica gel CC and eluted with gradient systems of Me_2_CO–CH_2_Cl_2_ and MeOH–Me_2_CO to yield nine fractions (C1–C9). Isolation of a precipitate obtained from fraction C3 (254.8 mg) by reverse phase silica gel C-18 CC using 50% MeOH in DI water as an eluent and followed by recrystallization from MeOH/CH_2_Cl_2_ produced compound **6** (gallic acid) (86.8 mg) as white needles. Fraction C4 (613.7 mg) was separated over reverse phase silica gel C-18 CC and eluted with 50% MeOH in DI water to afford seven fractions (D1–D7). The recrystallization fraction D3 from MeOH/CH_2_Cl_2_ yield compound **7** (myricitrin, myricetin 3-*O*-α-L-rhamnopyranoside) (129.8 mg) as a yellow powder. Fraction D6 (83.6 mg) was rechromatographed using reverse phase silica gel C-18 CC and eluted with 50% MeOH in DI water to provide six fractions (E1–E6). Compounds **8** (quercitrin, quercetin 3-*O*-α-L-rhamnopyranoside) (24.7 mg) was obtained as a yellow powder by recrystallization of subfraction E4 from MeOH/CH_2_Cl_2_. (See more details of CC in supplementary material)

### 3.5. Anticholinesterase Assay

Bioassay-guided separation of the leaf extracts based on acetylcholinesterase inhibitory activity was evaluated by TLC bioautographic assay according to Ellman’s method as previously described [53]. The leaf extracts and fractions were dissolved in suitable solvents. The sample solutions were separated by silica gel TLC developed with a suitable mobile phase. The TLC plates were sprayed with the solutions of acetylthiocholine iodide (ATCI) (30 mM) follow by 5,5′-dithiobis(2-nitrobenzoic acid) (DTNB) (20 mM) in Tris-HCl 50 mM, pH 8. After left for 5 min, the TLC plates were sprayed with enzyme acetylcholinesterase (AChE) solution (10 U/mL in Tris-HCl 50 mM, pH 8). The compounds corresponding to potential acetylcholinesterase inhibitors were appeared as white spots against a yellow background. The samples were determined for their inhibitory effect on acetylcholinesterase by a microtiterplate spectroscopic assay based on Ellman’s method [53,54]. The samples solutions were prepared at least 5 concentrations (conc.) by dissolving the solutes in 50% DMSO–20% tween 20 or 50% MeOH in buffer (50 mM Tris-HCl, pH 8.0 containing 0.1% BSA). Then, 25 µL of the sample solutions were added into 96-well microtiter plates and mixed with 50 µL of buffer, 125 µL of 3 mM DTNB in buffer containing 0.1 M NaCl and 0.02 M MgCl_2_.2H_2_O, and 25 µL of 15 mM ATCI in deionized water. The reaction mixtures were analyzed in triplicate by recording their absorbance at a wavelength of 405 nm with an Infinite M200 microplate reader (Tecan, Männedorf, Switzerland). After 25 µL of AChE solution (0.22 U/mL AChE in buffer containing 0.1% BSA) was added, the absorbance was measured again every 45 s for 5 cycles. Galanthamine was used as a positive control. The Percentage inhibition was calculated by using the equation: % AChE inhibition = [(A_control_ − A_sample_)/A_control_] × 100. The IC_50_ values of the samples were calculated by using linear regression analysis.

### 3.6. DPPH Radical-Scavenging Assay

Screening for antioxidant activity of the leaf extracts was carried out based on TLC bioautographic DPPH assay according to [46]. After separation of the extracts on a silica gel TLC plate developed with a suitable mobile phase, the TLC plates were sprayed with 0.2% DPPH solution in MeOH. The components which have antioxidant effect were detected as yellow spots against a purple background of the TLC plates. The concentration that could inhibit the DPPH radical formation by 50% (IC_50_) of the leaf extracts and isolated compounds were evaluated using a spectrophotometric method as previously described [46]. Five concentrations in 80% MeOH of the test samples were analyzed in triplicate by mixing 100 µL of each sample solutions and 0.02% DPPH methanolic solution in 96-well microtiter plates. After incubation of the reaction mixtures in the dark at room temperature for 10 min, the absorbance measurements were performed at 517 nm using a microplate reader (Tecan Infinite M200). Ascorbic acid and 6-hydroxy-2,5,7,8-tetramethylchroman-2-carboxylic acid (Trolox^®^) were used as positive controls. The DPPH radical scavenging activity was evaluated as percent inhibition which was calculated by the equation: % Inhibition = ((A_control_ − A_sample_) / A_control_) × 100.

### 3.7. Cell Culture

HEK-293 cells were purchased from American Type Culture Collection (ATCC; CRL-1573) and grown in DMEM with 10% FBS and 1% (*v/v*) penicillin/streptomycin solution at 37 °C in a 5% CO_2_ humidified atmosphere as previously described [46].

### 3.8. Determination of Cell Cytotoxicity by MTT Assay

Cell viability was assessed by MTT assay [55]. MeOH extract, myricitrin (**7**), quercitrin (**8**), were dissolved in DMSO at concentrations of 20 mg/mL (MeOH extract) and 50 mM (flavonoid glycosides **7**, **8**) as the stock solution. The stock solution was diluted with serum-free DMEM for making the desired concentrations. HEK-293 cells were cultured in 96-well plates (1 × 10^4^ cells/well) in DMEM supplemented with 1% FBS and allowed to adhere to plate’s bottom for overnight. Cells were then treated with methanol extract, myricitrin (**7**), quercitrin (**8**), or vehicle (DMSO) at various concentrations for 36 h. At the end of treatment, cells were incubated with MTT solution (2 mg/mL) for at least 4 h. After that, DMSO was added in each well to dissolve the purple formazan crystals. The absorbance was measured at a wavelength of 570 nm using a microplate reader (Tecan Infinite M200). The relative amounts of viable cells are directly proportional with the absorbance values. The results were shown as the percentage of cell viability (% Cell viability = [A_treated cells_/A_control cells_] × 100).

### 3.9. Measurement of ROS Production in HEK-293 Cells

The effects of the MeOH extract and some isolated compounds, myricitrin (**7**) and quercitrin (**8**) on inhibition of H_2_O_2_-induced ROS production were investigated in order to determine their intracellular antioxidant activities. The intracellular ROS levels were quantified by a fluorescent probe dichlorodihydrofluorescein diacetate (DCFH-DA) as previously described [42]. HEK-293 cells in 12-well plate (5 × 10^4^ cells/well in 1 mL of DMEM) were treated with the MeOH extract (50 and 100 µg/mL), the flavonoid glycosides **7**, **8** (10 μM), ascorbic acid (50 μM), or DMSO (control; vehicle) for 3 h. Cells were then treated with H_2_O_2_ (100 µM) for 1 h. After washing with phosphate buffered saline (PBS), cells were added with DCFH-DA (10 µM) in the dark for 30 min. The intracellular ROS levels are related to the DCF fluorescence intensity which were measured using a fluorescence microplate reader (BioTek, Winooski, VT, USA) at wavelength of 485/530 nm. Ascorbic acid was used as an antioxidant (positive control). The intracellular ROS production was expressed as the percentages of DCF-fluorescence intensities compared to the vehicle (DMSO) (% of ROS production = [DCF_sample_/DCF_vehicle_] × 100).

### 3.10. Determination of mRNA Expression

The mRNA level of antioxidant genes was analyzed by real-time quantitative reverse transcription polymerase chain reaction (qRT-PCR) as described previously [47]. HEK-293 cells were treated with DMSO (control), the MeOH extract (100 µg/mL), myricitrin (**7**) (10 μM), or quercitrin (**8**) (10 μM) for 6 h. After that, the total RNA was isolated by using RNA isolation kit (Thermo Scientific). The qRT-PCR reactions were performed with SYBR FAST One-step RT-qPCR kit (KAPA biosystems, Wilmington, DE, USA) and Mx 3005P qPCR system (Stratagene, La Jolla, CA, USA). The mRNA expression was calculated by using the comparative cycle threshold (CT) method and relative expression of targeted genes was normalized with GAPDH. The primers of antioxidant genes are shown in Table 2.

### 3.11. Statistical Analysis

Results are presented as mean ± SEM. All statistical analyses were assessed by SPSS software (version 25). Differences between groups were analyzed by one-way analysis of variance (ANOVA) with post hoc test. The value of p-value less than 0.05 (*p* < 0.05) was considered significant.

## 4. Conclusions

The present study demonstrates that the young leaves of *S. antisepticum* are benefit for health promotion and prevention of aging-associated diseases due to antioxidant and anticholinesterase activities. Triterpenoids and polyphenolic compounds were identified as the active components in the *S. antisepticum* young leaves. It was found that the indigenous vegetable and its flavonoids glycosides could suppress the intracellular ROS production and oxidative stress in the cells. Myricitrin and quercitrin found in the leaves of *S. antisepticum* exhibited cellular antioxidant defense mechanism by upregulating the synthesis of catalase and GPx-1 in HEK-293 cells. Moreover, ursolic acid and gallic acid was discovered as AChEIs in the plant extracts.

## Figures and Tables

**Table 1:** DPPH radical scavenging and anticholinesterase activities of the methanol extract and isolated compounds.

Compounds	DPPH Radical Scavenging		Anticholinesterase	
	% Inhibition	IC_50_ (µM)	% Inhibition	IC_50_ (µM)
Hexane extract	7.2 ± 2.33 ^1^	ND	ND ^4^	ND
Ethyl acetate extract	68.68 ± 1.42 ^1^	ND	ND ^4^	ND
Methanol extract	68.72 ± 0.32 ^1^	ND	61.90 ± 0.08 ^3^	ND
Betulinic acid (**1**)	15.90 ± 0.87 ^2^	ND	39.95 ± 1.53 ^2,5^	ND
Ursolic acid (**2**)	12.69 ± 1.33 ^2^	ND	73.39 ± 1.32 ^2,5^	159.32 ± 1.34 ^5^
Jacoumaric acid (**3**)	19.40 ± 2.51 ^2^	ND	29.73 ± 3.12 ^2,5^	ND
Corosolic acid (**4**)	3.61 ± 0.63 ^2^	ND	11.18 ± 0.30 ^2,6^	ND
Daucosterol (**5**)	4.11 ± 0.81 ^2^	ND	5.53 ± 1.47 ^2,6^	ND
Gallic acid (**6**)	95.80 ± 0.32 ^2^	14.46 ± 0.29	81.64 ± 0.29 ^2,6^	161.06 ± 9.52 ^6^
Myricitrin (**7**)	95.27 ± 0.45 ^2^	31.01 ± 0.90	10.91 ± 1.69 ^2,6^	ND
Quercitrin (**8**)	93.16 ± 0.11 ^2^	38.34 ± 0.74	10.21 ± 1.26 ^2,6^	ND
Ascorbic acid (Vitamin C)	ND	59.73 ± 2.73	ND	ND
Trolox	ND	50.10 ± 3.56	ND	ND
Galantamine ^5^	ND	ND	ND	14.76 ± 0.63 ^5^
Galantamine ^6^	ND	ND	ND	6.02 ± 0.42 ^6^

ND = not determine; ^1^ at conc. 50 µg/mL, ^2^ at conc. 100 µg/mL, ^3^ at conc. 300 µg/mL, ^4^ solubility problem, ^5^ dissolved with 50% DMSO–20% Tween 20 in buffer, ^6^ dissolved with 50% methanol in buffer.

**Table 2:** The specific primers for human antioxidant genes.

Gene Specific Primers		Sequences
Glutathione peroxidase-1 (GPx-1)	Sense Antisense	5′-ctcttcgagaagtgcgaggt-3′ 5′-tcgatgtcaatggtctggaa-3′
Catalase	Sense Antisense	5′-gcagatacctgtgaactgtc-3′ 5′-gtagaatgtccgcacctgag-3′
Heme oxygenase-1 (HO-1)	Sense Antisense	5′-caggcagagaatgctgag-3′ 5′-gcttcacatagcgctgca-3′
Manganese superoxide dismutase (Mn-SOD)	Sense Antisense	5′-gcacattaacgcgcagatca-3′ 5′-agcctccagcaactctcctt-3′
Glutathione reductase (GRe)	Sense Antisense	5′-cagtgggactcacggaagat-3′ 5′-ttcactgcaacagcaaaacc-3′
Glyceraldehyde 3-phosphate dehydrogenase (GAPDH)	Sense Antisense	5′-cgagatccctccaaaatcaa-3′ 5′-gtcttctgggtggcagtgat-3′

## Data Availability

Not applicable.

## Supplementary Materials

The following are available online. Spectroscopic and physical data of isolated compounds from *Syzygium antisepticum* Leaves, separation experiment detail.

## Sample Availability

Samples of the compounds are not available from the authors.
