# Supplementary material for: Antioxidant and Anticholinesterase Activities of Extracts and Phytochemicals of Syzygium antisepticum Leaves
Source: Molecules. 2021 May 30;26(11):3295. doi: 10.3390/molecules26113295 (PMC8198064; doi:10.3390/molecules26113295)
Supplement: Supplementary file 1 [file molecules-26-03295-s001.zip › molecules-1232880-supplementary.pdf]

Supplementary Materials

Antioxidant and Anticholinesterase Activities of Extracts and Phytochemicals of *Syzygium antisepticum* Leaves

Supachoke Mangmool [1], Issaree Kunpukpong [2], Worawan Kitphati [3] and Natthinee Anantachoke [2,4,*]

[1] Department of Pharmacology, Faculty of Science, Mahidol University, Bangkok 10400, Thailand; supachoke.man@mahidol.ac.th.
[2] Department of Pharmacognosy, Faculty of Pharmacy, Mahidol University, Bangkok 10400, Thailand; emmi.pornsiri@gmail.com.
[3] Department of Physiology, Faculty of Pharmacy, Mahidol University, Bangkok 10400, Thailand; worawan.kit@mahidol.ac.th.
[4] Center of Excellence for Innovation in Chemistry, Faculty of Science, Mahidol University, Bangkok 10400, Thailand
* Correspondence: natthinee.ana@mahidol.ac.th

Spectroscopic and physical data of isolated compounds from *Syzygium antisepticum* Leaves.

1. **Betulinic acid (1) (3*β*-Hydroxyl-19*β*-hydrogen-lup-20(29)-en-28-oic acid)**

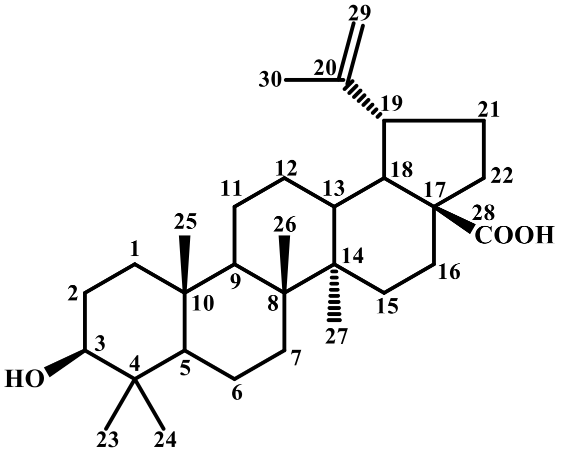

Physical characteristics: white needles from MeOH/CH_2Cl_2, m.p. 278.0-279.0°C

FTIR ν_{max}^{KBr} cm^{-1}: 3600-3200 (3451), 2942, 2869, 1687, 1639, 1579, 1453, 1376, 1321, 1299, 1236, 1188, 1108, 1044, 984, 972, 944, 887, 741

[α]$_D^{23}$: +5.16° (*c*1.0, pyridine)

EIMS *m/z* (% relative intensity): 456 [M]$^+$ (4), 411 [M-COOH] (8), 248 (39), 203 (55), 189 (100)

^{1}H-NMR (500 MHz, pyridine-*d*$_5$) δ_H *ppm* (mult., *J* (Hz)): 1.65 (1H, *dt*, 13.0, 3.3, H-1), 0.97 (1H, *m*, H-1), 1.85 (2H, *m*, H-2), 3.45 (1H, *dd*, 8.9, 7.2, H-3), 0.81 (1H, *m*, H-5), 1.54 (1H, *m*, H-6), 1.40 (1H, *m*, H-6), 1.39 (2H, *m*, H-7), 1.40 (1H, *m*, H-9), 1.42 (1H, *m*, H-11), 1.20 (1H, *m*, H-11), 1.93 (1H, *m*, H-12), 1.20 (1H, *m*, H-12), 2.73 (1H, *td*, 11.5, 3.3, H-13), 1.87 (1H, *m*, H-15), 1.25 (1H, *m*, H-15), 2.62 (1H, *br. dt*, 12.7, 3.3, H-16), 1.54 (1H, *m*, H-16), 1.75 (1H, *t*, 11.5, H-18), 3.53 (1H, *m*, H-19), 2.23 (1H, *m*, H-21), 1.54 (1H, *m*, H-21), 2.23 (1H, *m*, H-22), 1.54 (1H, *m*, H-22), 1.22 (3H, *s*, H-23), 1.00 (3H, *s*, H-24), 0.81 (3H, *s*, H-25), 1.05 (3H, *s*, H-26), 1.06 (3H, *s*, H-27), 4.94 (1H, *br. s*, H-29), 4.77 (1H, *br. s*, H-29), 1.78 (3H, *s*, H-30)

^{13}C-NMR (125 MHz, pyridine-*d*$_5$) δ_C *ppm*: 39.34 (C-1), 28.34 (C-2), 78.19 (C-3), 39.57 (C-4), 55.98 (C-5), 18.83 (C-6), 34.88 (C-7), 41.17 (C-8), 51.01 (C-9), 37.57 (C-10), 21.26 (C-11), 26.17 (C-12), 38.67 (C-13), 42.90 (C-14), 30.33 (C-15), 32.93 (C-16), 56.69 (C-17), 49.82 (C-18), 47.82 (C-19), 151.38 (C-20), 31.26 (C-21), 37.63 (C-22), 28.72 (C-23), 16.39 (C-24), 16.47 (C-25), 16.47 (C-26), 14.95 (C-27), 178.93 (C-28), 110.00 (C-29), 19.53 (C-30)

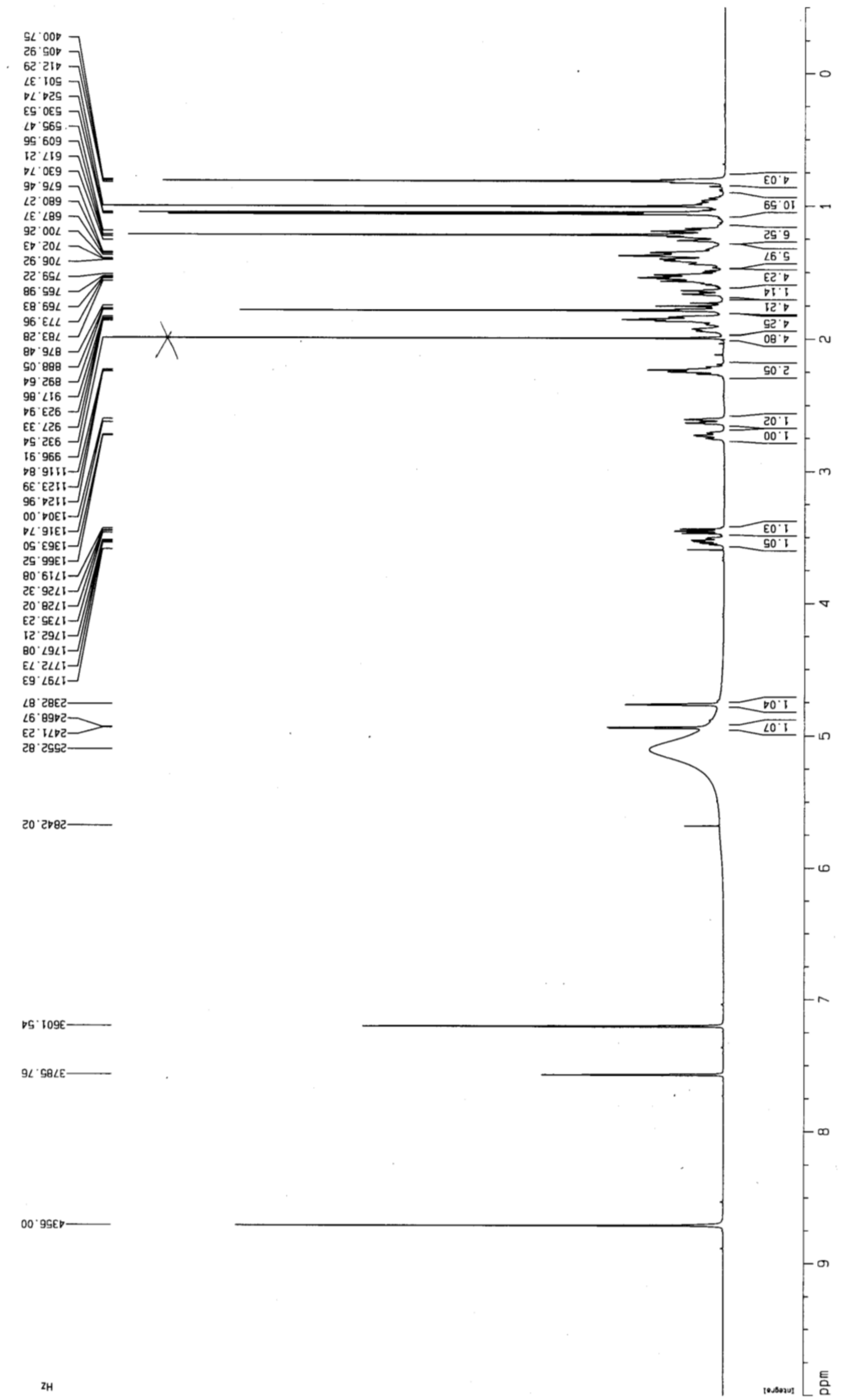

^{1}H-NMR spectrum of betulinic acid (500 MHz, pyridine-d_5)

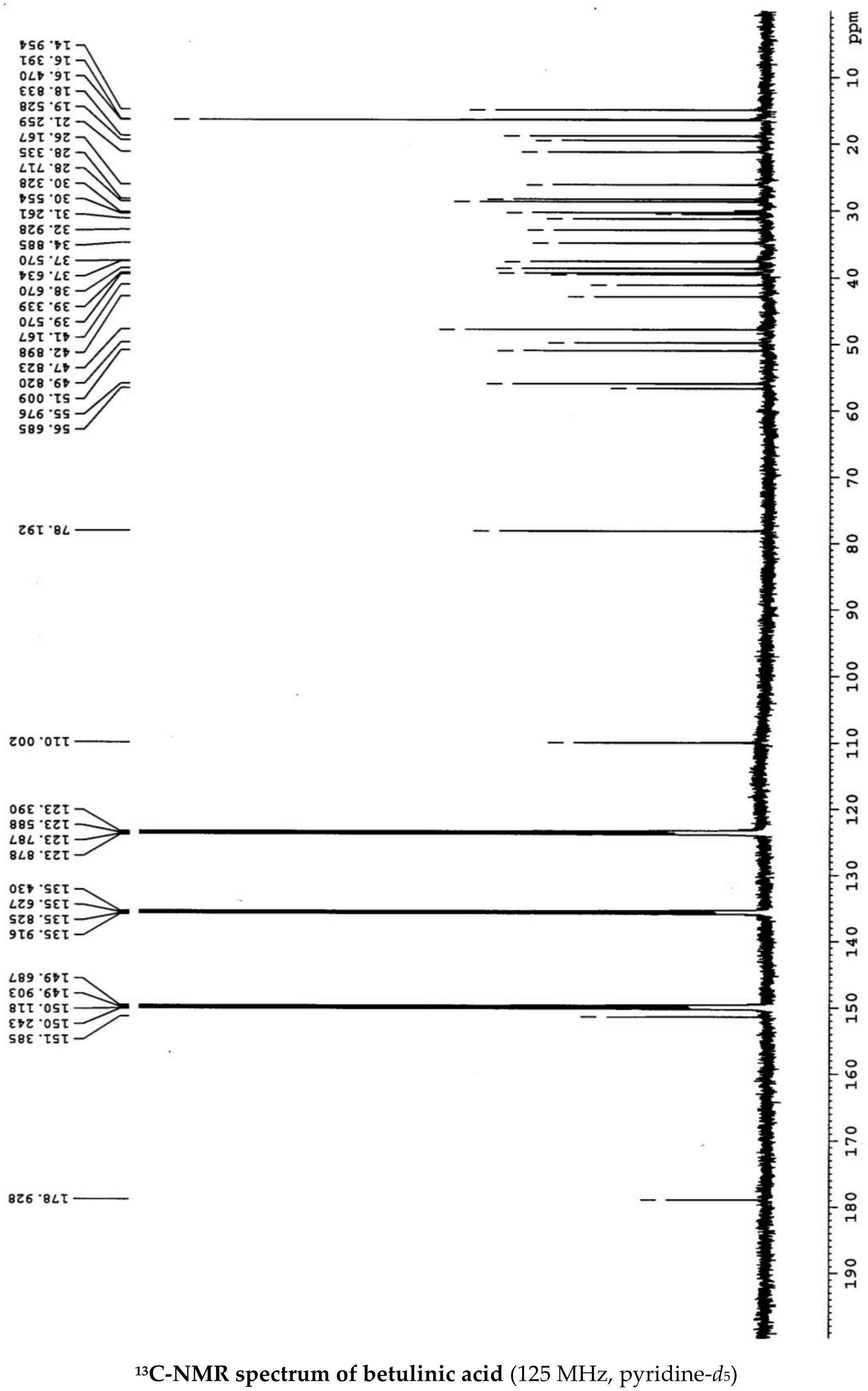

^{13}C-NMR spectrum of betulinic acid (125 MHz, pyridine-d_5)

2. Ursolic acid (2) (3β-Hydroxy-urs-12-ene-28-oic-acid)

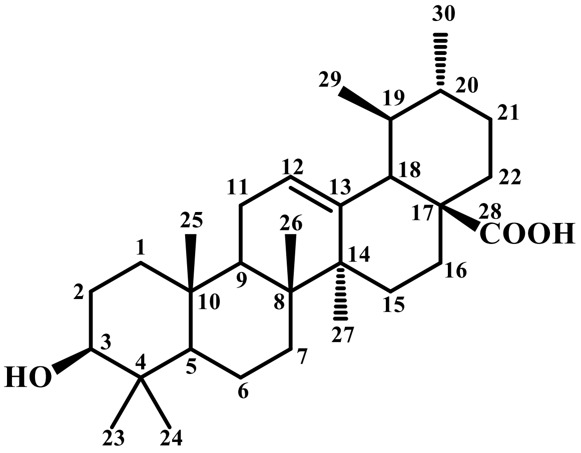

Physical characteristics: white powder from MeOH/CH_2Cl_2, m.p. 238.0-240.0°C

FTIR ν_{max}^{KBr} cm^{-1}: 3600-3200 (3432), 2950, 2871, 1690, 1457, 1388, 1285, 1028, 998.

[α] $_D^{24}$: +40.1° (c 0.5, methanol)

EIMS ***m/z*** (% relative intensity): 456 $[M]^+$ (2), 248 (100) (calcd. for $C_{16}H_{24}O_2$: 248.1770), 219 (24), 203 (71), 189 (22), 161 (16), 133 (2), 119 (18), 91(26), 55 (11)

^{1}H-NMR (500 MHz, DMSO-d_6) δ_H *ppm* (mult., *J* (Hz)): 1.51 (1H, *m*, H-1), 0.90 (1H, *m*, H-1), 1.45 (2H, *m*, H-2), 2.99 (1H, *m*, H-3), 0.65 (1H, *m*, H-5), 1.45 (1H, *m*, H-6), 1.28 (1H, *m*, H-6), 1.43 (1H, *m*, H-7), 1.25 (1H, *m*, H-7), 1.45 (1H, *m*, H-9), 1.84 (2H, *m*, H-11), 5.11 (1H, *t*, 3.0, H-12), 1.79 (1H, *m*, H-15), 0.98 (1H, *m*, H-15), 1.90 (1H, *m*, H-16), 1.51 (1H, *m*, H-16), 2.09 (1H, *d*, 11.0, H-18), 1.51 (1H, *m*, H-19), 0.97 (1H, *m*, H-20), 1.43 (2H, *m*, H-21), 1.53 (2H, *m*, H-22), 0.88 (3H, *s*, H-23), 0.73 (3H, *s*, H-24), 0.85 (3H, *s*, H-25), 0.66 (3H, *s*, H-26), 1.02 (3H, *s*, H-27), 0.79 (3H, *d*, 6.4, H-29), 0.98 (3H, *d*, 9.8, H-30)

^{13}C-NMR (125 MHz, DMSO-d_6) δ_C *ppm*: 38.26 (C-1), 26.98 (C-2), 76.87 (C-3), 38.38 (C-4), 54.81 (C-5), 18.00 (C-6), 32.72 (C-7), 41.65 (C-8), 47.04 (C-9), 36.33 (C-10), 22.86 (C-11), 124.58 (C-12), 138.20 (C-13), 41.65 (C-14), 27.55 (C-15), 23.82 (C-16), 46.84 (C-17), 52.40 (C-18), 38.46 (C-19), 38.55 (C-20), 30.20 (C-21), 36.53 (C-22), 28.26 (C-23), 16.10 (C-24), 15.22 (C-25), 17.00 (C-26), 23.27 (C-27), 178.28 (C-28), 16.92 (C-29), 21.10 (C-30)

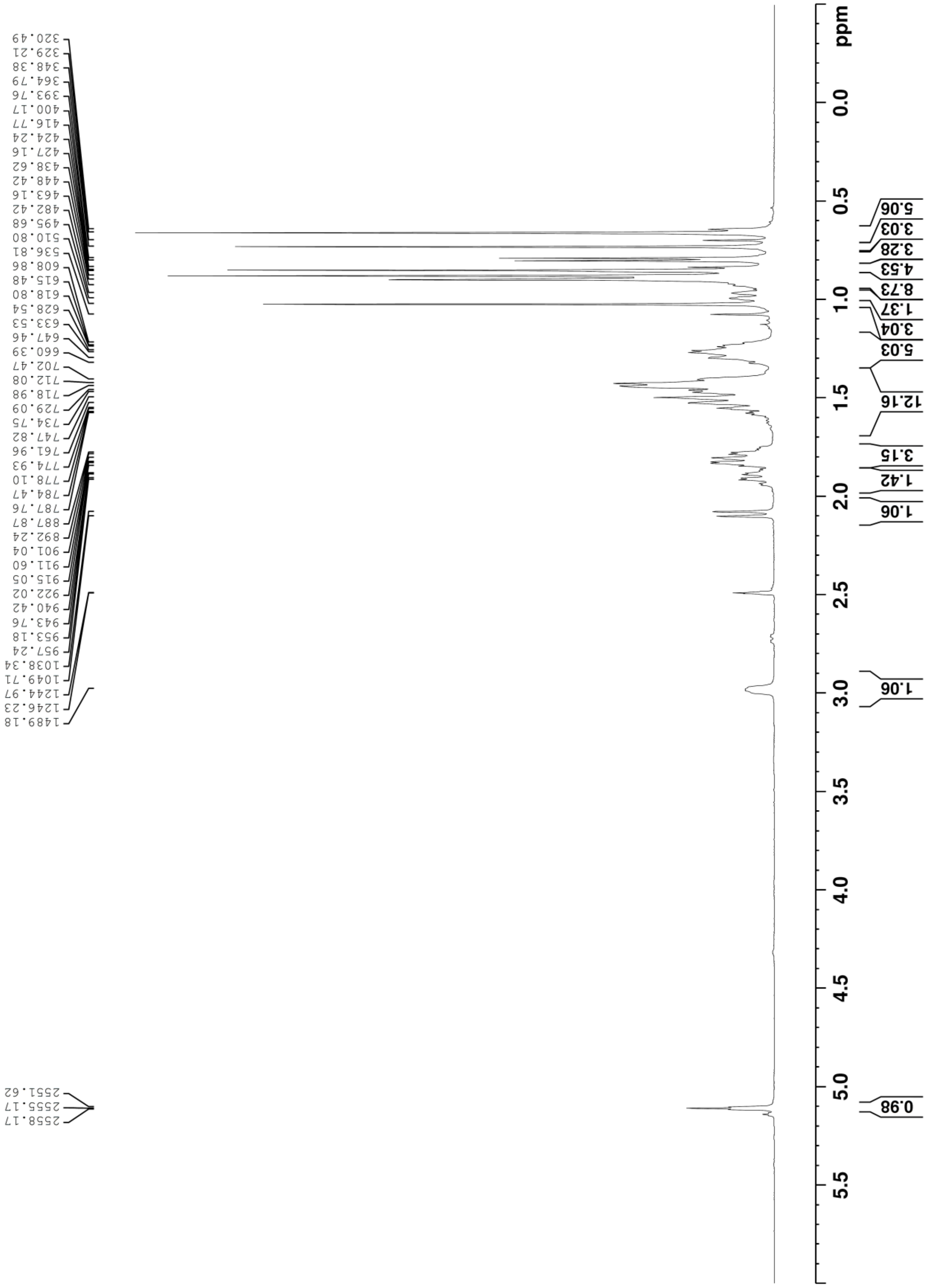

^{1}H-NMR spectrum of ursolic acid (500 MHz, DMSO-d_6, water suppression)

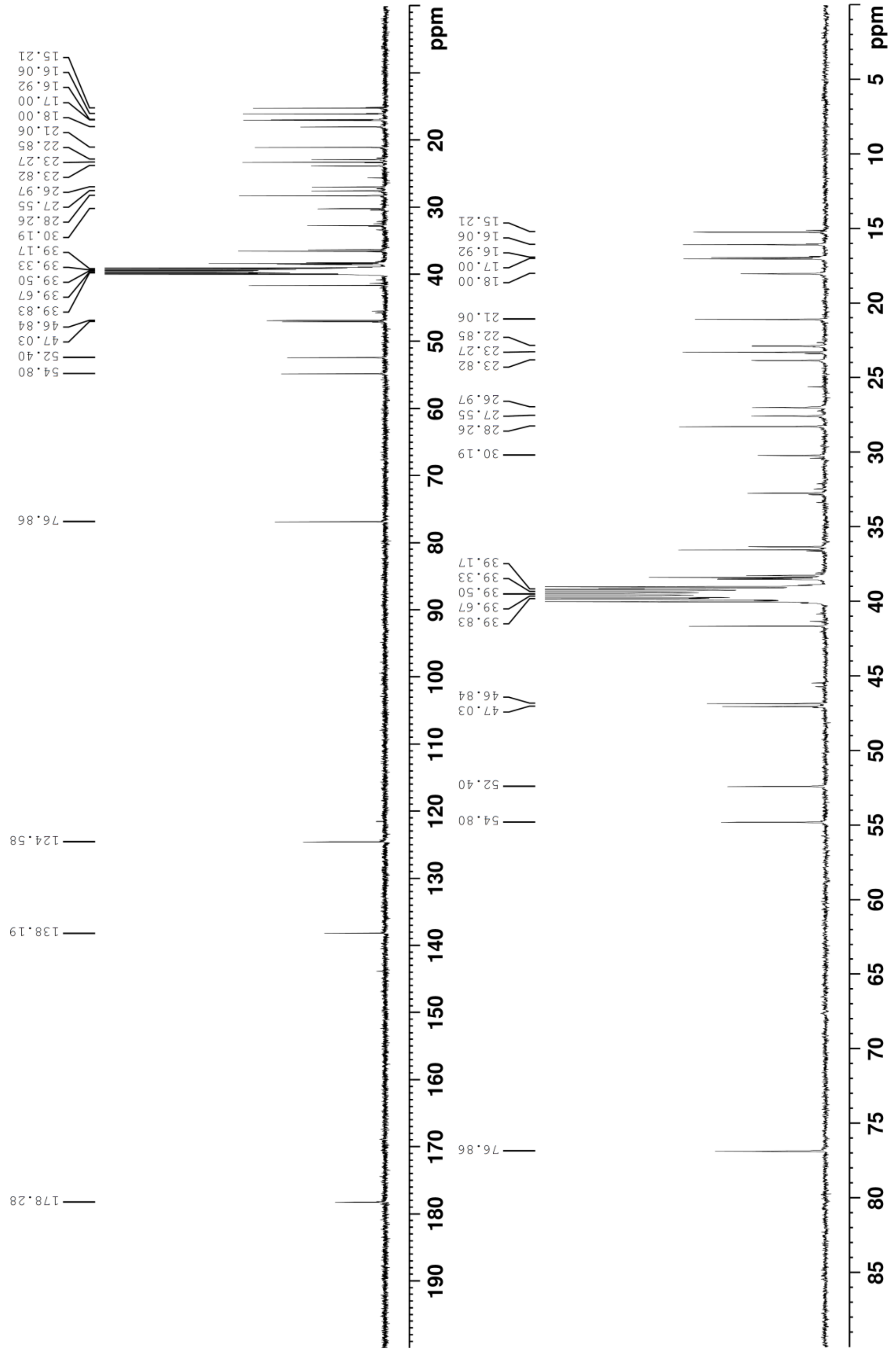

^{13}**C-NMR spectrum of ursolic acid** (125 MHz, DMSO-d_6)

3. **Jacoumaric acid (3) (2*α*-Hydroxy-3*β*-*trans*-*p*-coumaryloxy-urs-12-en-28-oic acid)**

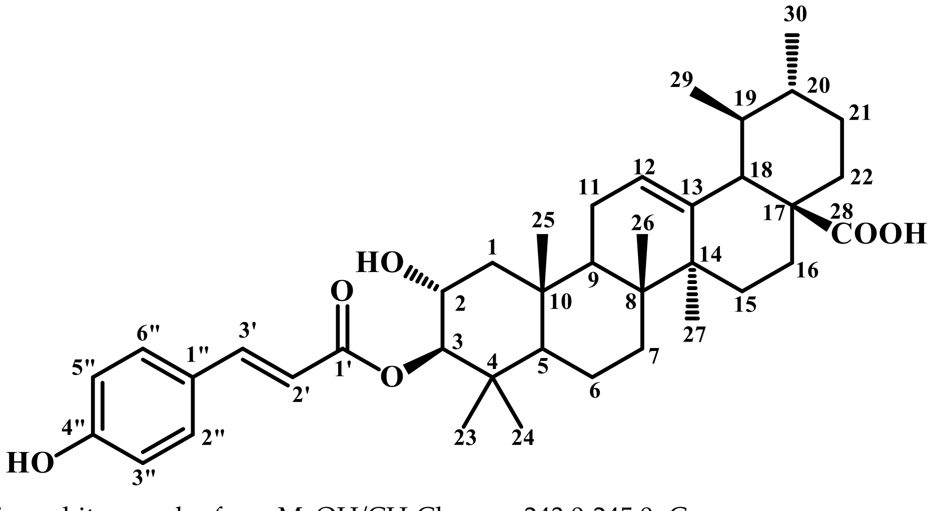

Physical characteristics: white powder from MeOH/CH_2Cl_2, m.p. 243.0-245.0 °C

FTIR ν_{max}^{KBr} cm^{-1}: 3550-3100 (3325), 2970, 2944, 2870, 1695, 1634, 1606, 1515, 1455, 1368, 1310, 1268, 1202, 1169, 1108, 1045, 982, 960, 944, 830

UV λ_{max}^{MeOH} **nm** (log *ε*): 209 (sh, 4.48), 225 (4.41), 301 (sh, 4.58), 312 (4.62),

$[\alpha]_D^{24}$: +13.3 ° (*c* 0.5, methanol)

EIMS *m/z* (% relative intensity): 454 (5) [M^+-$C_9H_7O_2$], 409 (5), 248 (40), 133 (100)

^{1}H-NMR (500 MHz, DMSO-d_6) δ_H *ppm* (mult., *J* (Hz)): 1.87 (1H, *m*, H-1), 0.96 (1H, *m*, H-1), 3.68 (1H, *m*, H-2), 4.51 (1H, *d*, 9.0, H-3), 0.91 (1H, *m*, H-5), 1.45 (1H, *m*, H-6), 1.35 (1H, *m*, H-6), 1.25 (2H, *m*, H-7), 1.55 (1H, *m*, H-9), 1.87 (1H, *m*, H-11), 1.84 (1H, *m*, H-11), 5.14 (1H, *br t*, 3.4, H-12), 1.78 (1H, *m*, H-15), 0.98 (1H, *m*, H-15), 1.93 (1H, *m*, H-16), 1.53 (1H, *m*, H-16), 2.11 (1H, *d*, 11.3, H-18), 0.91 (1H, *m*, H-19), 0.91 (1H, *m*, H-20), 1.41 (2H, *m*, H-21), 1.57 (1H, *m*, H-22), 1.53 (1H, *m*, H-22), 0.79 (3H, *s*, H-23), 0.84 (3H, *s*, H-24), 0.96 (3H, *s*, H-25), 0.74 (3H, *s*, H-26), 1.06 (3H, *s*, H-27), 0.82 (3H, *d*, 6.3, H-29), 0.91 (3H, *s*, H-30), 6.38 (1H, *d*, 15.9, H-2′), 7.52 (1H, *d*, 15.9, H-3′), 7.53 (1H, *d*, 8.8, H-2″), 6.78 (1H, *d*, 8.8, H-3″), 6.78 (1H, *d*, 8.8, H-5″), 7.53 (1H, *d*, 8.8, H-6″), 4.54 (1H, *d*, 5.6, 2-OH), 10.00 (1H, *s*, 4″-OH)

^{13}C-NMR (125 MHz, DMSO-d_6) δ_C *ppm*: 47.62 (C-1), 64.78 (C-2), 83.61 (C-3), 39.00 (C-4), 54.39 (C-5), 17.88 (C-6), 32.47 (C-7), 41.71 (C-8), 46.87 (C-9), 37.48 (C-10), 22.94 (C-11), 124.35 (C-12), 138.31 (C-13), 41.71 (C-14), 27.48 (C-15), 23.78 (C-16), 46.87 (C-17), 52.36 (C-18), 38.40 (C-19), 38.48 (C-20), 30.15 (C-21), 36.29 (C-22), 28.47 (C-23), 17.83 (C-24), 16.38 (C-25), 16.88 (C-26), 23.23 (C-27), 178.25 (C-28), 17.02 (C-29), 21.05 (C-30), 166.65 (C-1′), 115.11 (C-2′), 143.96 (C-3′), 125.24 (C-1″), 130.09 (C-2″), 115.75 (C-3″), 159.60 (C-4″), 115.75 (C-5″), 130.09 (C-6″)

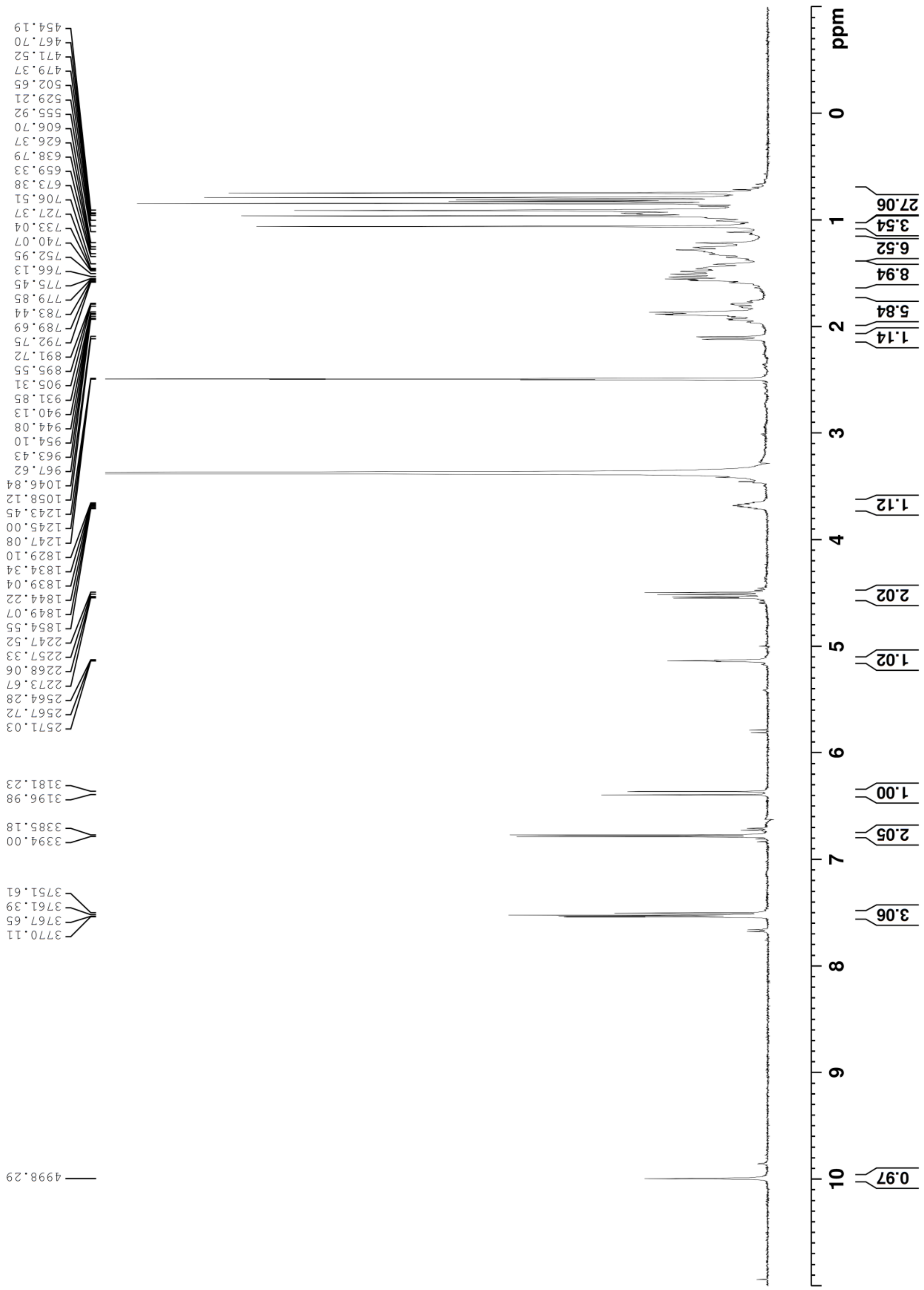

^{1}H-NMR spectrum of jacoumaric acid (125 MHz, DMSO-*d_6*)

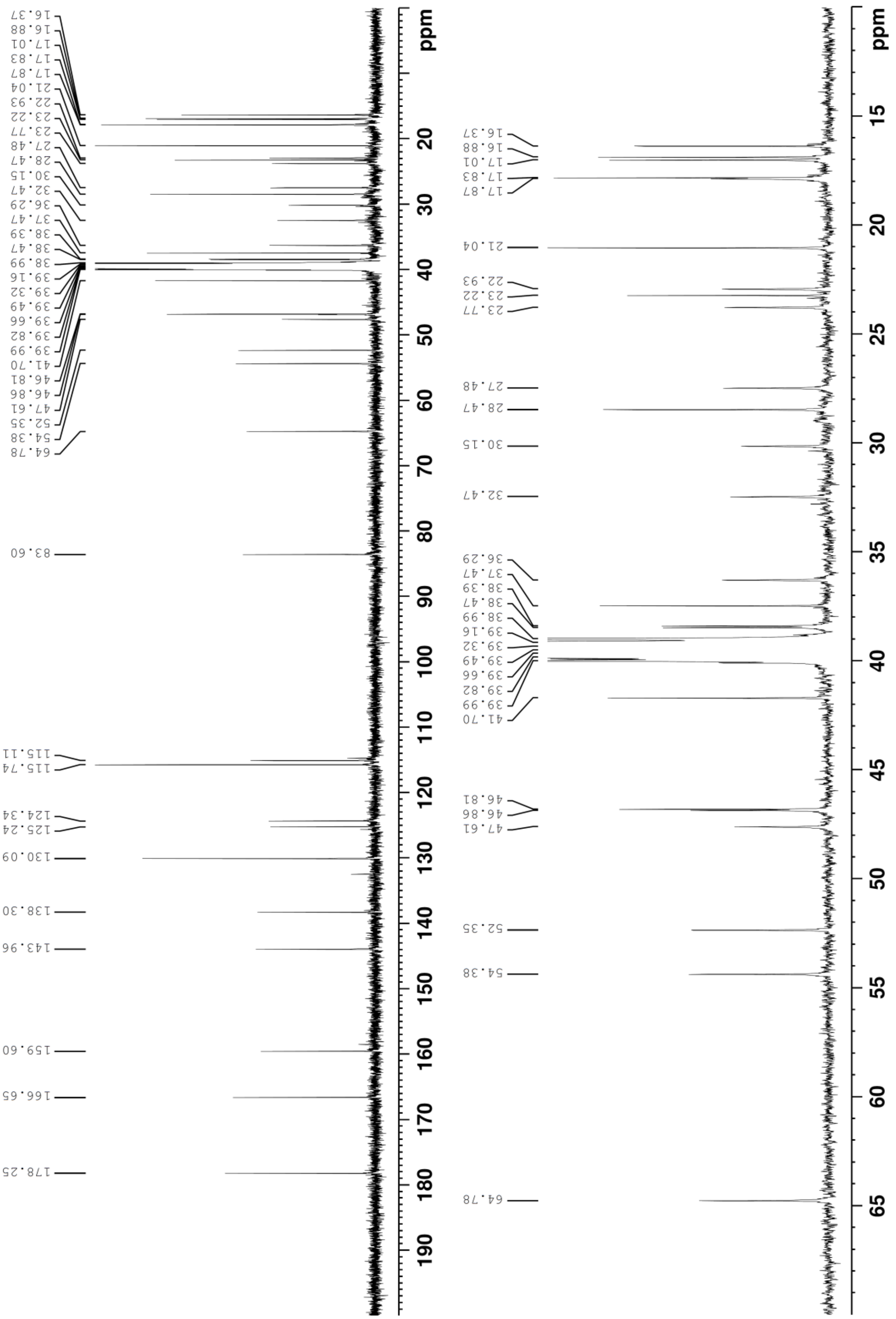

^{13}C-NMR spectrum of jacoumaric acid (500 MHz, DMSO-d_6)

4. **Corosolic acid (4) (2*α*, 3*β*-Dihydroxy-urs-12-ene-28-oic-acid)**

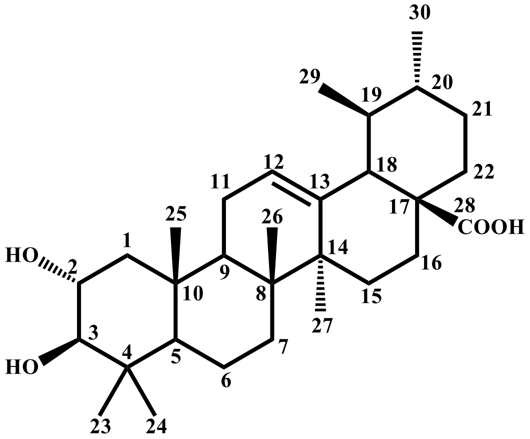

Physical characteristics: white powder from MeOH/CH_2Cl_2, m.p. 238.1-240.3°C

FTIR ν_{max}^{KBr} cm^{-1}: 3600-3200 (3448), 2927, 1701, 1459, 1378, 1243, 1050

[α] $_{D}^{23}$: +16.75° (c 1.0, methanol)

EIMS *m/z*: 248 (100), 219 (32), 203 (75), 133 (87)

^{1}H-NMR (500 MHz, DMSO-d_6) δ_H *ppm* (mult., *J* (Hz)): 1.76 (1H, *m*, H-1), 0.75 (1H, *m*, H-1), 3.46 (1H, *m*, H-2), 2.72 (1H, *d*, 9.4, H-3), 0.74 (1H, *m*, H-5), 1.44 (1H, *m*, H-6), 1.30 (1H, *m*, H-6), 1.44 (1H, *m*, H-7), 1.27 (1H, *m*, H-7), 1.49 (1H, *m*, H-9), 1.85 (2H, *dd*, 6.9, 3.5, H-11), 5.12 (1H, *br t*, 3.5, H-12), 0.97 (1H, *br d*, 13.4, H-15), 1.91 (1H, *m*, H-16), 1.52 (1H, *m*, H-16), 2.09 (1H, *d*, 11.1, H-18), 0.90 (1H, *m*, H-19), 0.90 (1H, *m*, H-20), 1.42 (2H, *m*, H-21), 1.56 (1H, *m*, H-22), 1.50 (1H, *m*, H-22), 0.90 (3H, *s*, H-23), 0.72 (3H, *s*, H-24), 0.90 (3H, *s*, H-25), 0.68 (3H, *s*, H-26), 1.02 (3H, *s*, H-27), 0.80 (3H, *d*, 6.4, H-29), 0.90 (3H, *br s*, H-30)

^{13}C-NMR (125 MHz, DMSO-d_6) δ_C *ppm*: 47.10 (C-1), 67.24 (C-2), 82.33, (C-3), 39.83 (C-4), 54.80 (C-5), 18.08 (C-6), 32.69 (C-7), 41.76 (C-8), 47.05 (C-9), 37.60 (C-10), 23.00 (C-11), 124.54 (C-12), 138.32 (C-13), 41.76 (C-14), 27.56 (C-15), 23.86 (C-16), 46.90 (C-17), 52.45 (C-18), 38.50 (C-19), 38.56 (C-20), 30.24 (C-21), 36.83 (C-22), 28.88 (C-23), 17.06 (C-24), 16.48 (C-25), 17.01 (C-26), 23.33 (C-27), 178.41 (C-28), 17.22 (C-29), 21.13 (C-30)

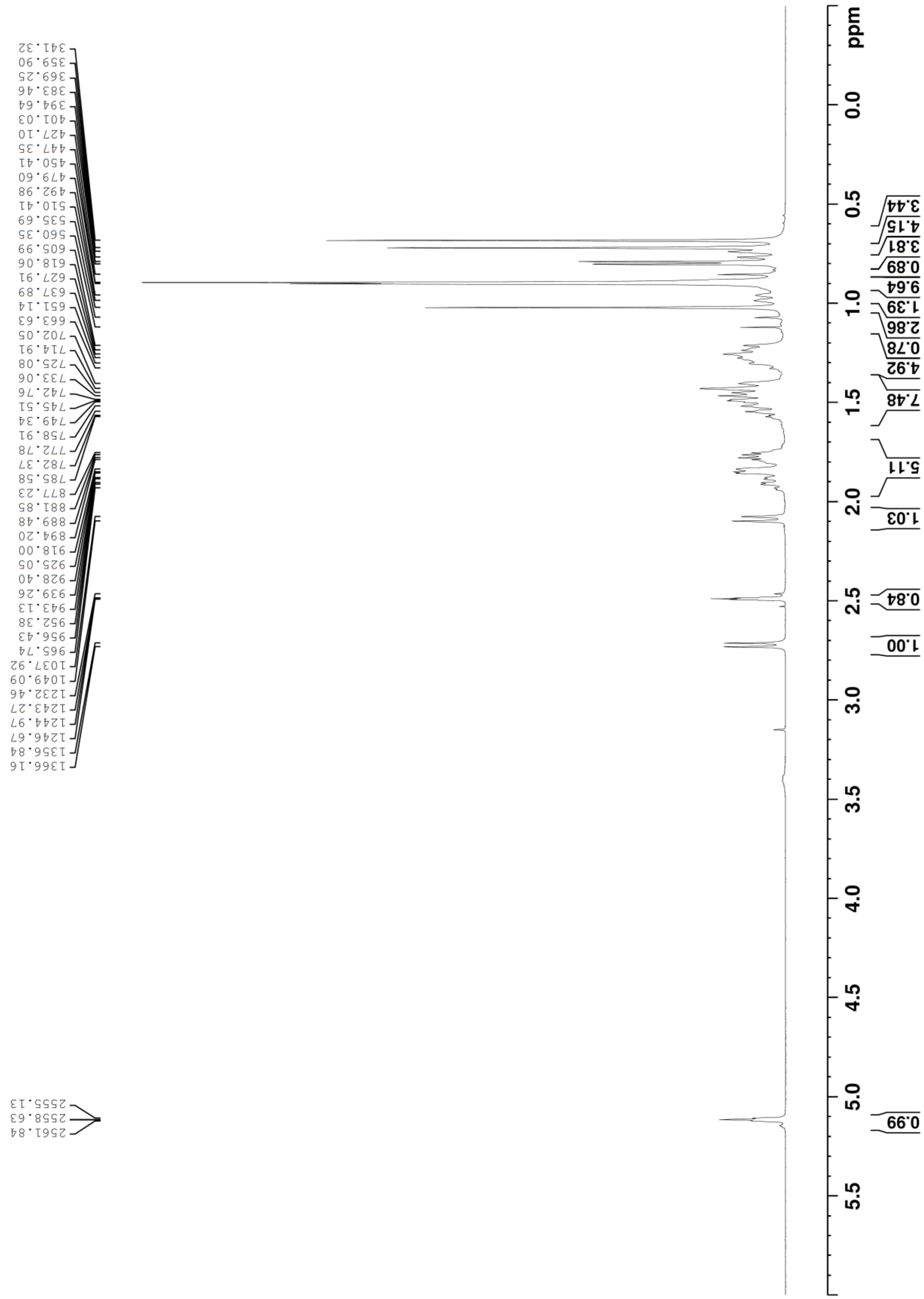

^{1}H-NMR spectrum of corosolic acid (500 MHz, DMSO-d_6, water suppression)

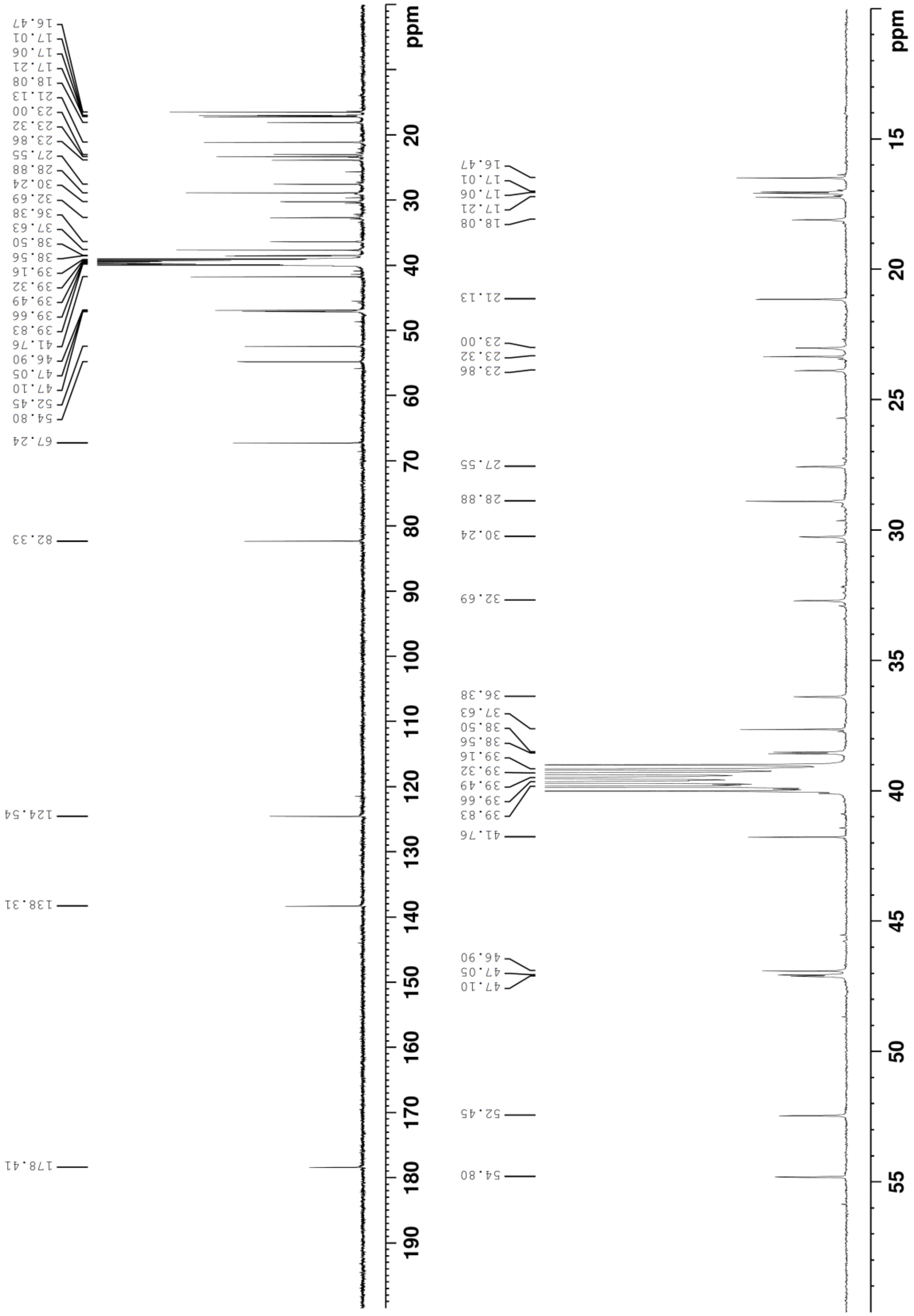

^{13}C-NMR spectrum of corosolic acid (125 MHz, DMSO-d_6)

5. **Daucosterol (5) (Stigma-5-en-3-*O*-*β*-glucoside)**

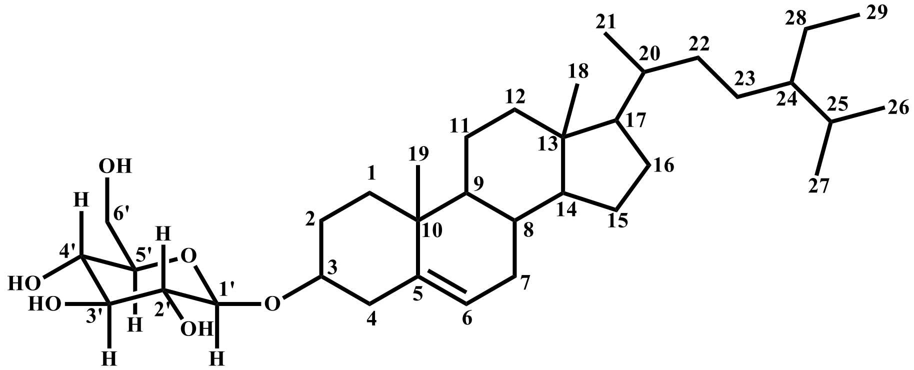

Physical characteristics: white powder from MeOH/CH_2Cl_2, m.p. 280-285.0 °C

FTIR ν_{max}^{KBr} cm^{-1}: 3600-3200 (3411), 2950, 2920, 1636, 1465, 1367, 1074, 1024

[α] $_{D}^{23}$: -48.37(c 1, MeOH)

EIMS *m/z* (% relative intensity): 576.53 $[M]^+$ (2), 414.36 (9), 397.40 (76), 396.34 (100), 381.39 (26), 255.25 (27)

^{1}H-NMR (500 MHz, pyridine-d_5) δ_H *ppm* (mult., *J* (Hz)): 1.00 (1H, *m*, H-1), 1.76 (1H, *m*, H-1), 1.76 (1H, *m*, H-2), 2.16 (1H, br *d*, 10.7, H-2), 3.98 (1H, *m*, H-3), 2.50 (1H, *br t*, 11.1, H-4), 2.76 (1H, *br d*, 11.1, H-4), 5.38 (1H, *br s*, H-6), 1.69 (1H, *m*, H-7), 1.91 (1H, *m*, H-7), 1.39 (1H, *m*, H-8), 0.90 (1H, *m*, H-9), 1.45 (2H, *m*, H-11), 1.13 (1H, *m*, H-12), 2.00 (1H, *m*, H-12), 0.98 (1H, *m*, H-14), 1.06 (1H, *m*, H-15), 1.57 (1H, *m*, H-15), 1.27 (1H, *m*, H-16), 1.86 (1H, *m*, H-16), 1.13 (1H, *m*, H-17), 0.68 (1H, *s*, H-18), 0.96 (1H, *s*, H-19), 1.41 (1H, *m*, H-20), 1.01 (3H, *d*, 6.3, H-21), 1.11 (1H, *m*, H-22), 1.41 (1H, *m*, H-22), 1.27 (2H, *m*, H-23), 1.01 (1H, *m*, H-24), 1.70 (1H, *m*, H-25), 0.88 (3H, *d*, 6.4, H-26), 0.90 (3H, *d*, 6.4, H-27), 1.32 (2H, *m*, H-28), 0.93 (3H, *t*, 7.5, H-29), 5.07 (1H, *d*, 7.7, H-1′), 4.09 (1H, *br t*, 7.6, H-2′), 4.31 (1H, *m*, H-3′), 4.31 (1H, *m*, H-4′), 4.01 (1H, *m*, H-5′), 4.43 (1H, *dd*, 11.2, 4.7, H-6′), 4.59 (1H, *br d*, 11.2, H-6′)

^{13}C-NMR (125 MHz, pyridine-d_5) δ_C *ppm*: 37.58 (C-1), 30.33 (C-2), 78.30 (C-3), 39.43 (C-4), 141.04 (C-5), 121.97 (C-6), 32.26 (C-7), 32.17 (C-8), 50.46 (C-8), 37.02 (C-10), 21.38 (C-11), 40.06 (C-12), 42.59 (C-13), 56.95 (C-14), 24.59 (C-15), 28.61 (C-16), 56.37 (C-17), 12.07 (C-18), 19.50 (C-19), 36.46 (C-20), 19.11 (C-21), 34.32 (C-22), 26.55 (C-23), 46.16 (C-24), 29.61 (C-25), 19.32 (C-26), 20.05 (C-27), 23.51 (C-28), 12.25 (C-29), 102.65 (C-1′), 75.37 (C-2′), 78.45 (C-3′), 71.80 (C-4′), 78.63 (C-5′), 62.92 (C-6′)

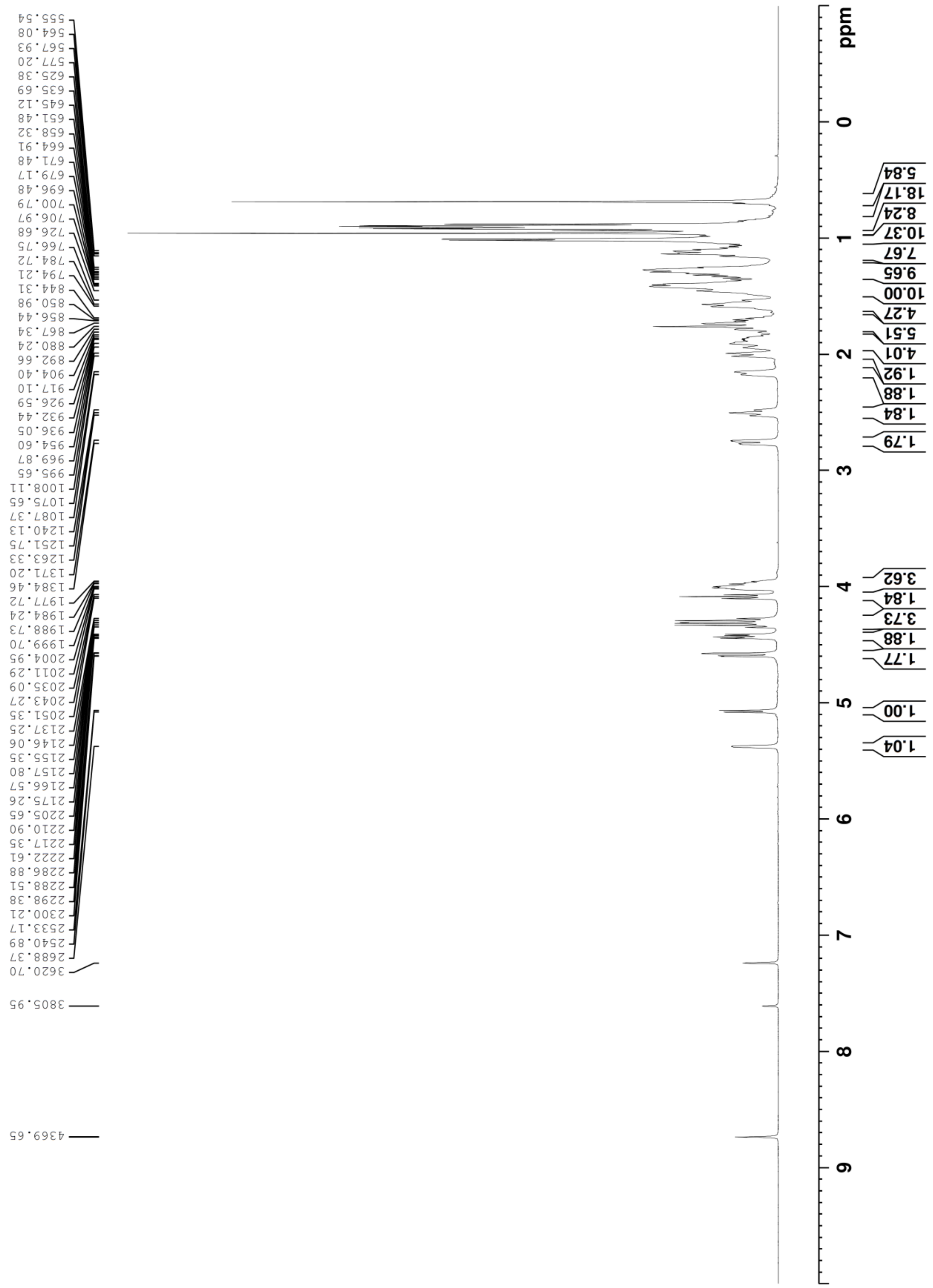

^{1}H-NMR spectrum of daucosterol (500 MHz, pyridine-d_5, water suppression)

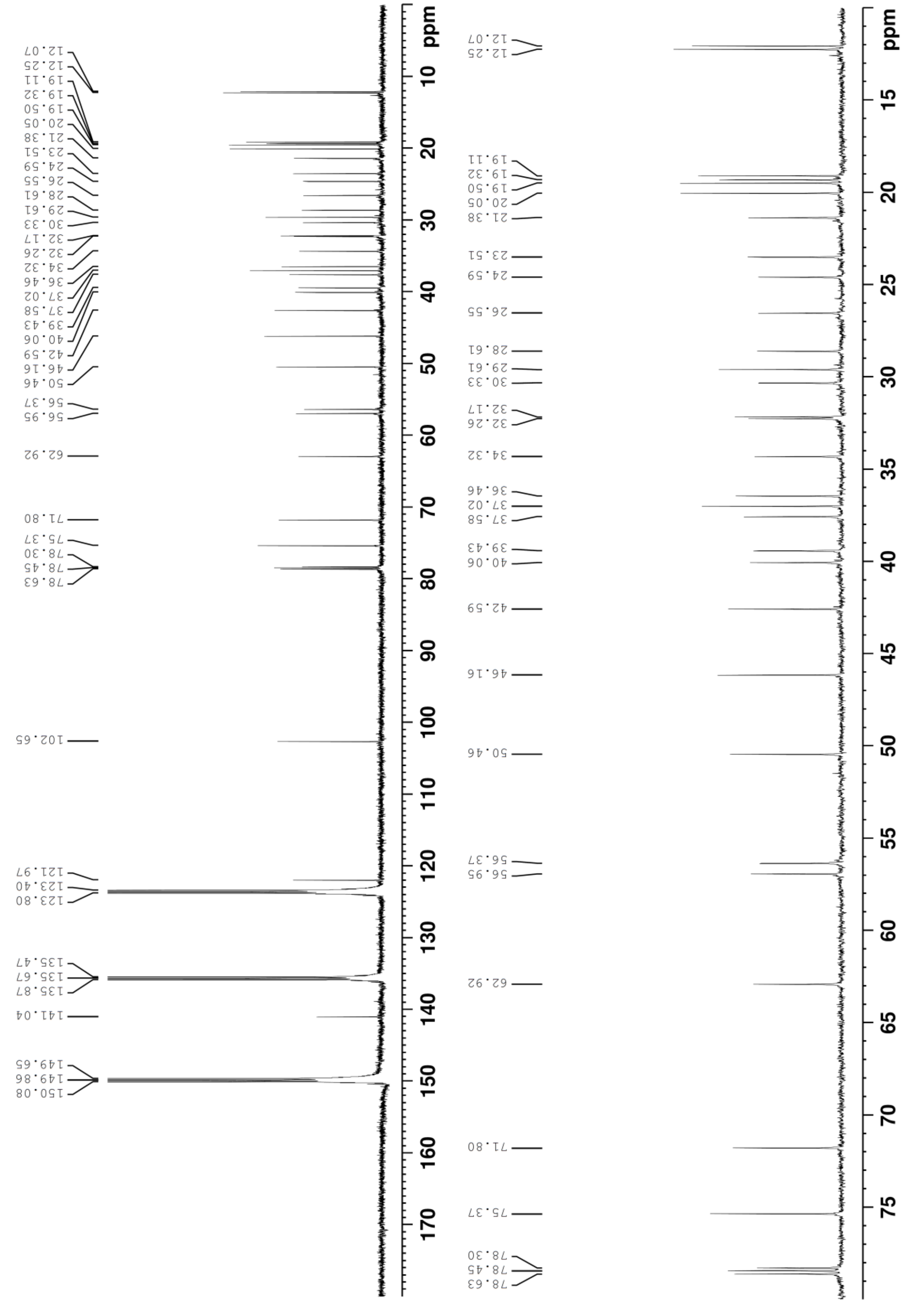

^{13}C-NMR spectrum of daucosterol (125 MHz, pyridine-*d*5)

6. Gallic acid (6)

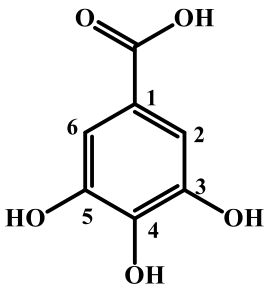

Physical characteristics: white needles from MeOH/CH_2Cl_2, m.p. 258.7°C

UV λ_{max}^{MeOH} nm (log *ε*): 270.40 (4.08), 227.60 (4.14)

FTIR ν_{max}^{KBr} cm^{-1}: 3400, 3286, 1701, 1617, 1541, 1450,1339, 1247, 1026, 866

EIMS *m/z* (% relative intensity): 170 $[M]^+$ (100), 149 (97), 125 $[M^+-CO_2]$ (27)

HR-ESI-MS *m/z*: 193.0139 $[M+Na]^+$

^{1}H-NMR (500 MHz, DMSO-d_6) δ_H *ppm* (mult., *J* (Hz)): 6.93 (2H, *s*, H-2, H-6)

^{13}C-NMR (125 MHz, DMSO-d_6) δ_C *ppm*: 119.35 (C-1), 108.54 (C-2, C-6), 145.59 (C-3, C-5), 138.42 (C-4), 166.35 (1-CO)

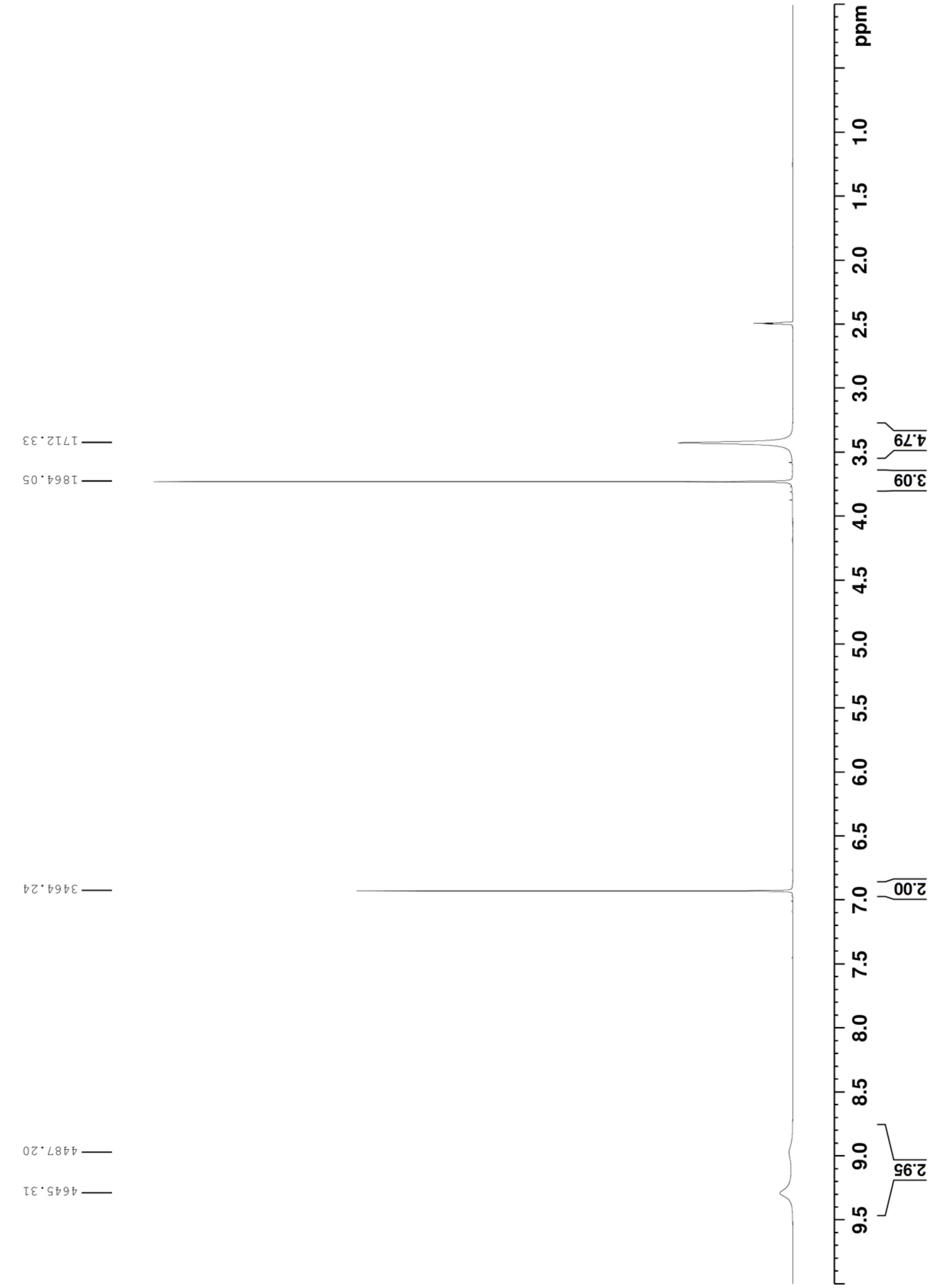

[1]**H-NMR spectrum of gallic acid** (500 MHz, DMSO-*d*$_6$, water suppression)

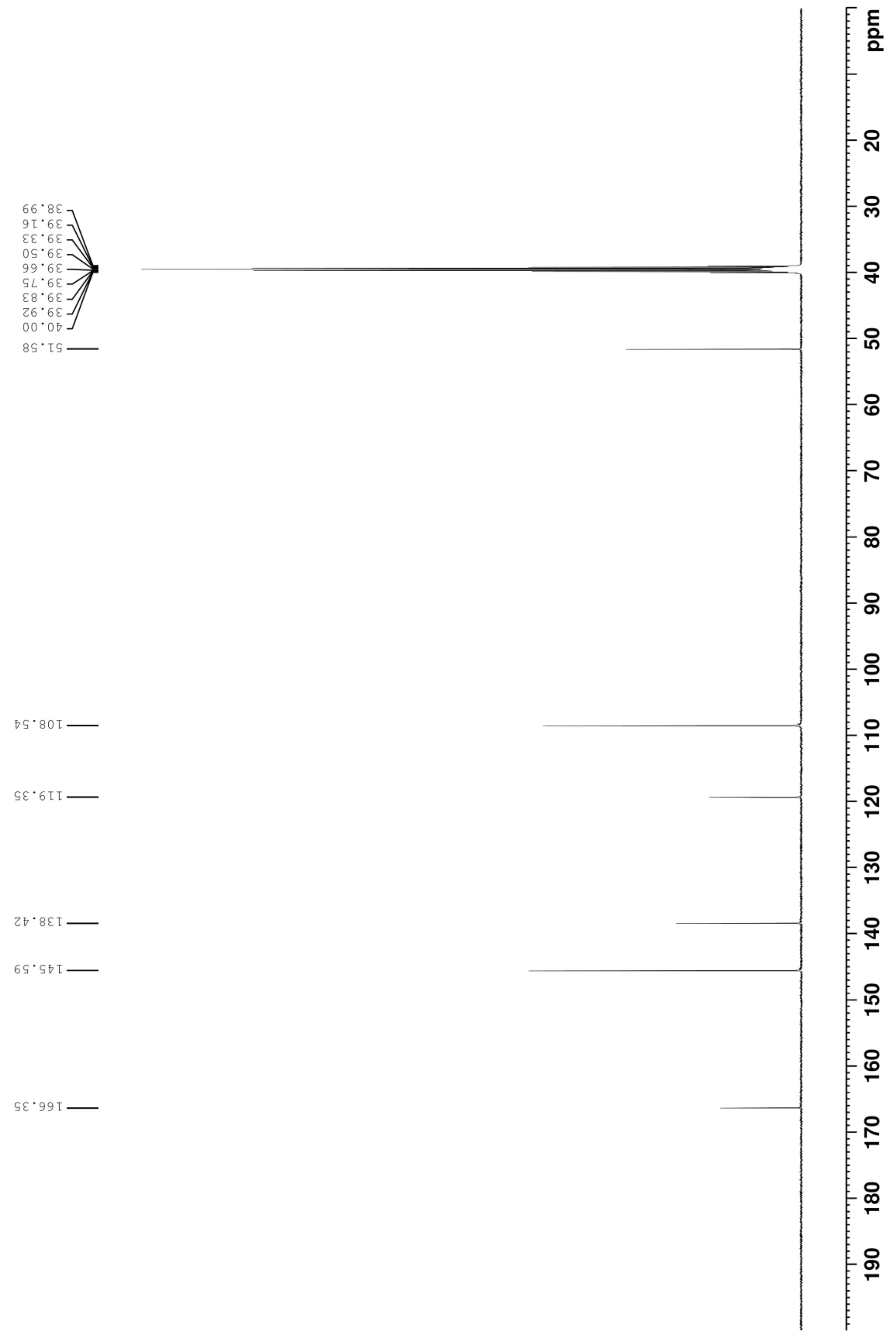

^{13}C-NMR spectrum of gallic acid (125 MHz, DMSO-d_6)

7. Myricitrin (7) (Myricetin 3-*O*-*α*-L-rhamnopyranoside)

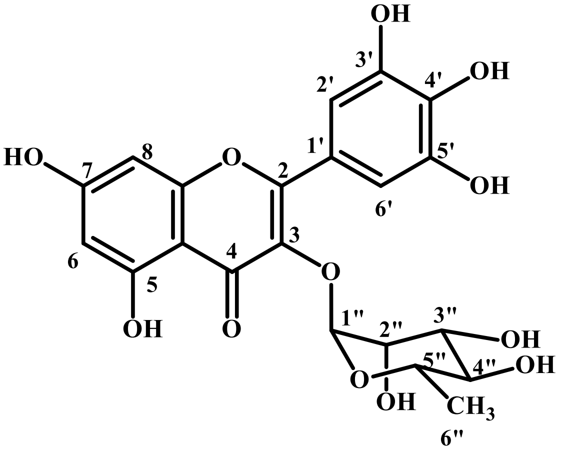

Physical characteristics: yellow powder from MeOH/CH_2Cl_2, m.p. 220.1-221.7°C

UV λ_{max}^{MeOH} nm (log *ε*): 353 (5.29), 235 (5.21)

FTIR ν_{max}^{KBr} cm^{-1}: 3600-3400 (3386), 1654, 1604, 1499, 1453, 1345, 1291, 1198, 1164, 1039, 968

$[\alpha]_D^{23}$: -126.16 (c 1.0, methanol) (lit $[\alpha]_D^{25}$: -120.0° (c 0.6, methanol)) [27]

EIMS *m*/*z* (% relative intensity): 466 $[M+H]^+$ (1), 318 [M^+-rhamnose] (100), 153 (25)

^{13}C-NMR (125 MHz, DMSO-*d*$_6$) δ_C *ppm*: 156.42 (C-2), 134.30 (C-3), 177.80 (C-4), 161.32 (C-5), 98.66 (C-6), 164.17 (C-7), 93.53 (C-8), 157.50 (C-9), 104.06 (C-10), 119.64 (C-1′), 107.93 (C-2′), 145.77 (C-3′), 136.46 (C4′), 145.77 (C-5′), 107.93 (C-6′), 101.95 (C-1″), 70.02 (C-2″), 70.40 (C-3″), 71.30 (C-4″), 70.55 (C-5″), 17.60 (C-6″)

^{1}H-NMR (500 MHz, DMSO-*d*$_6$) δ_H *ppm* (mult., *J* (Hz)): 6.36 (1H, *d*, 1.9, H-8), 6.85 (1H, *s*, H-2′), 6.85 (1H, *s*, H-6′), 5.18 (1H, *s*, H-1″), 3.97 (1H, *s*, H-2″), 3.54 (1H, *d*, 7.5, H-3″), 3.24 (1H, *t*, 9.5, H-4″), 3.34 (1H, *m*, H-5″), 0.82 (1H, *d*, 6.1, H-6″), 12.68 (1H, *s*, 5-OH)

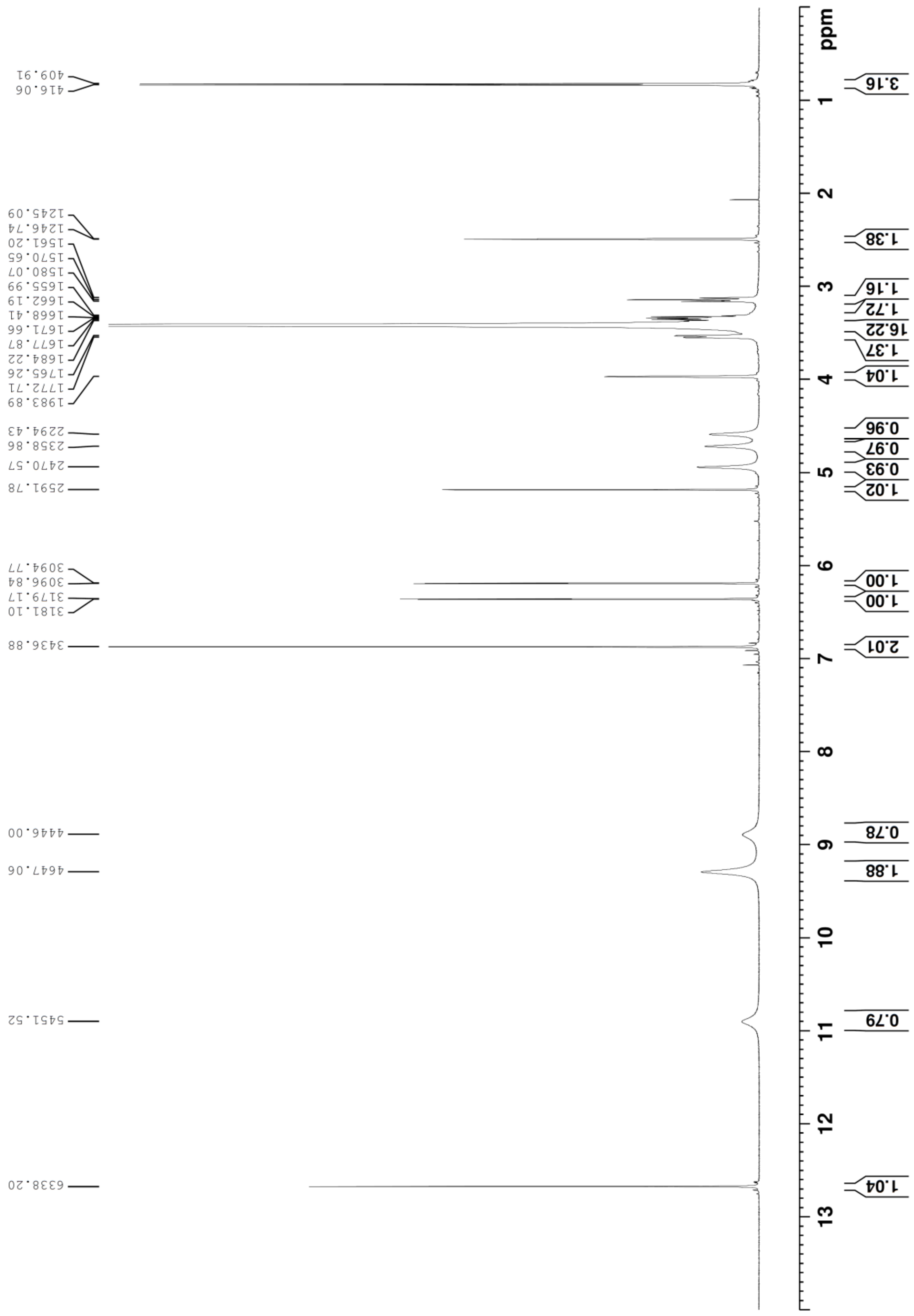

^{1}H-NMR spectrum of myricitrin (500 MHz, DMSO-d_6)

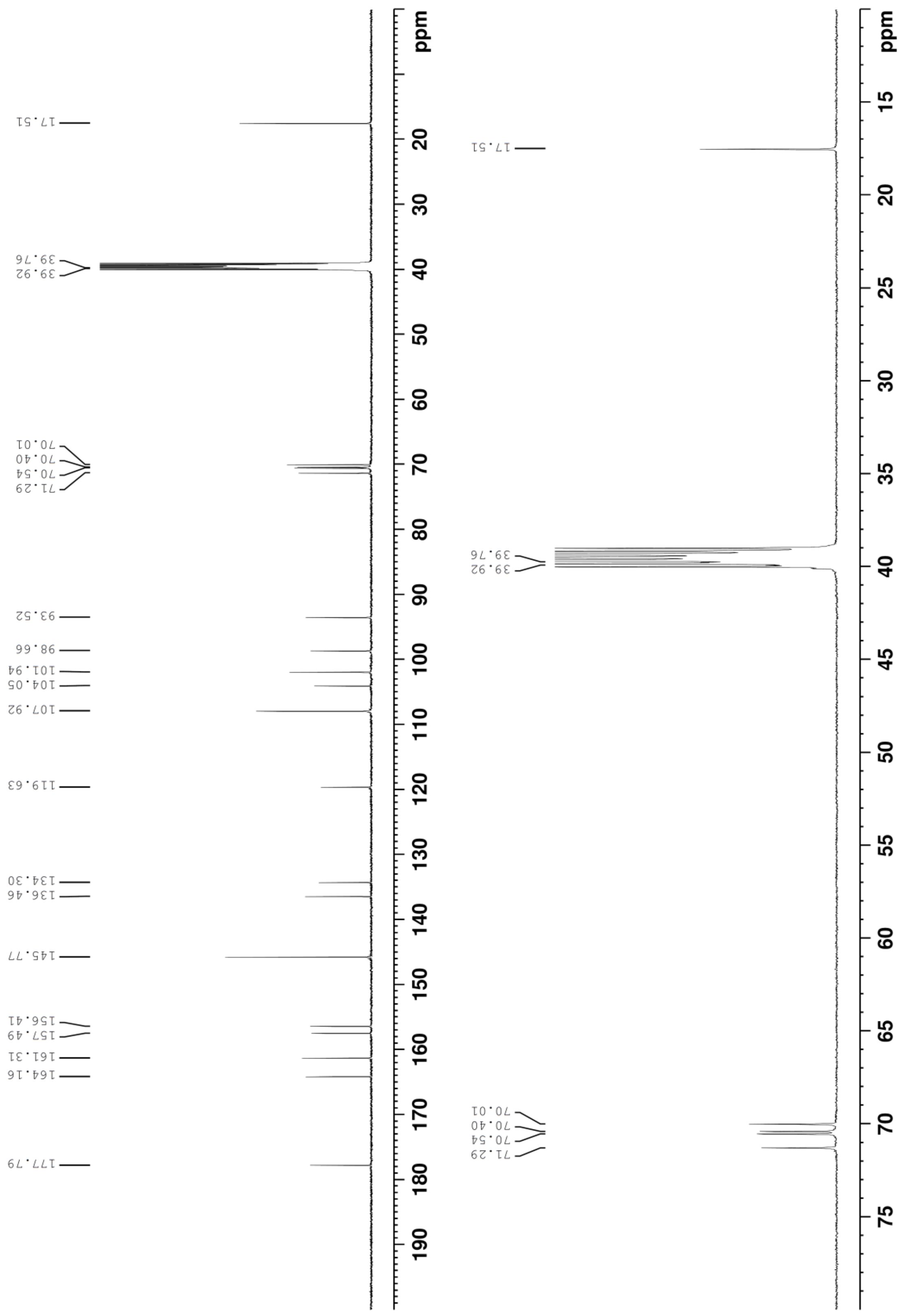

^{13}C-NMR spectrum of myricitrin (125 MHz, DMSO-d_6)

8. Quercitrin (8) (Quercetin 3-*O*-*α*-L-rhamnopyranoside)

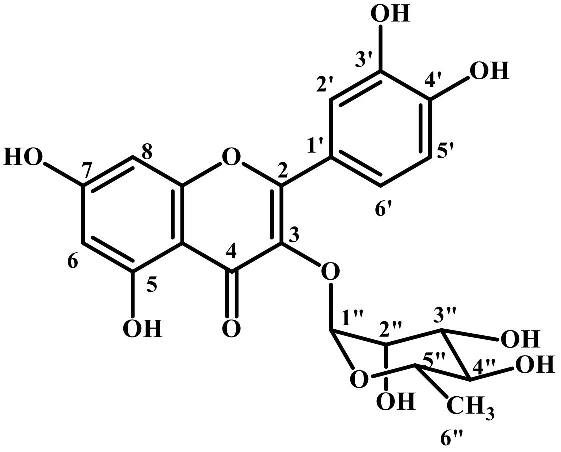

Physical characteristics: yellow powder from MeOH/CH_2Cl_2 m.p. 174.0-176.0°C

UV λ_{max}^{MeOH} nm (log *ε*): 350 (5.65), 253 (5.73).

FTIR ν_{max}^{KBr} cm^{-1}: 3600-3400 (3428), 1653, 1610, 1507, 1383, 1203

[α]$_D^{23}$: -31.28 ° (c 1, methanol) ([α]$_D^{25}$: -147.0 ° (c 0.5, methanol)) [30]

EIMS *m*/*z* (% relative intensity): 449 $[M+H]^+$ (0.45), 302 [M-rhamnose] (100)

^{13}C-NMR (125 MHz, methanol-*d*4) δ_C *ppm*: 158.54 (C-2), 136.25 (C-3), 179.66 (C-4), 163.21 (C-5), 99.85 (C-6), 165.91 (C-7), 94.74 (C-8), 159.31 (C-9), 105.91 (C-10), 123.02 (C-1′), 116.39 (C-2′), 146.41 (C-3′), 149.79 (C-4′), 116.98 (C-5′), 122.87 (C-6′), 103.55 (C-1″), 72.02 (C-2″), 72.17 (C-3″), 73.30 (C-4″), 71.91 (C-5″), 17.64 (C-6″)

^{1}H-NMR (500 MHz, methanol-*d*4) δ_H *ppm* (mult., *J* (Hz)): 6.19 (1H, *d*, 2.0, H-6), 6.36 (1H, *d*, 2.0, H-8), 7.33 (1H, *d*, 2.1, H-2′), 6.90 (1H, *d*, 8.3, H-5′), 7.30 (1H, *dd*, 8.3, 2.1, H-6′), 5.34 (1H, *d*, 1.6, H-1″), 4.21 (1H, *dd*, 3.28, 1.6, H-2″), 3.74 (1H, *dd*, 9.4, 3.4, H-3″), 3.41 (1H, *m*, H-4″), 3.33 (1H, *m*, H-5″), 0.93 (3H, *d*, 6.1, H-6″)

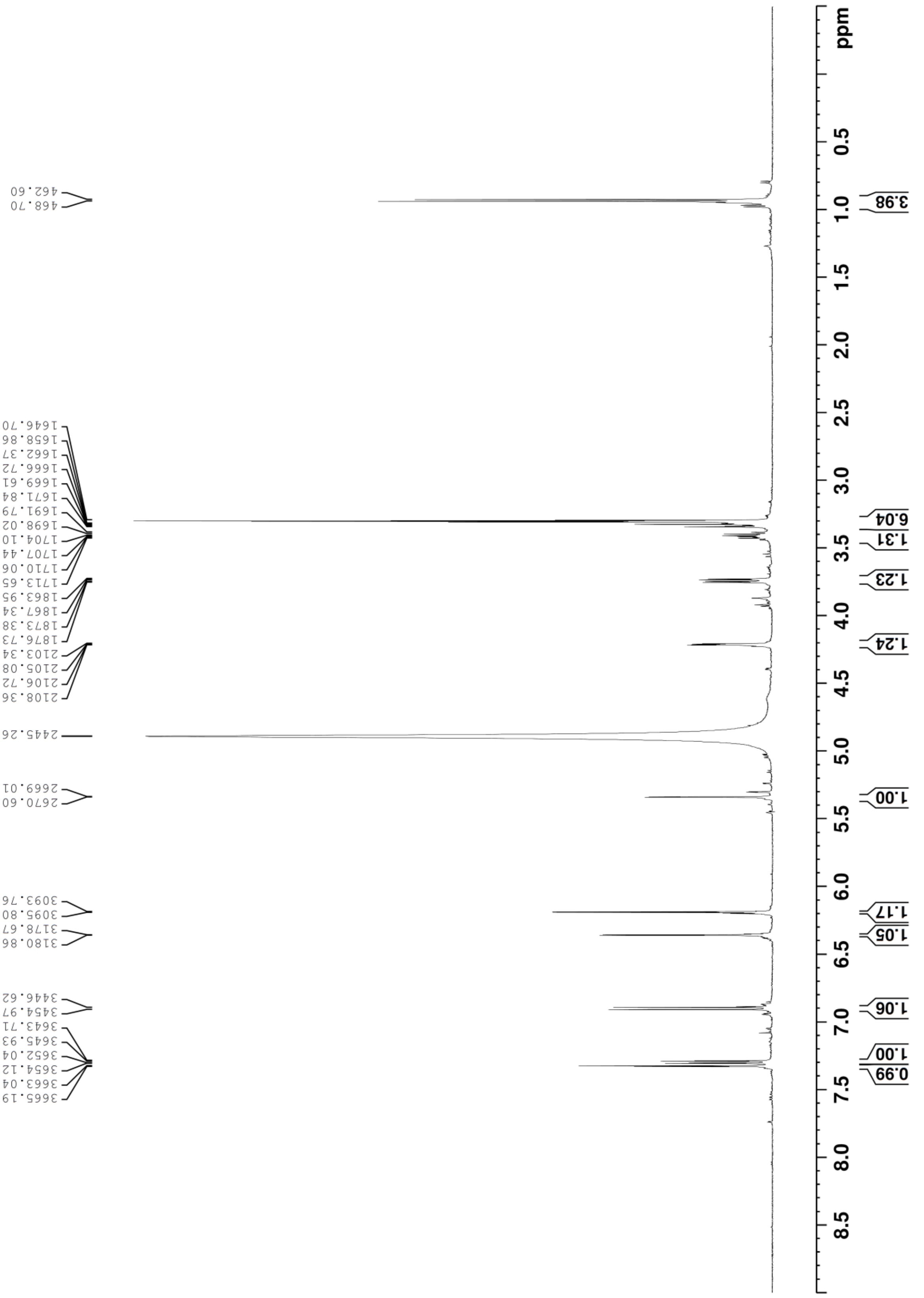

^{1}H-NMR spectrum of quercitrin (500 MHz, methanol-d_4)

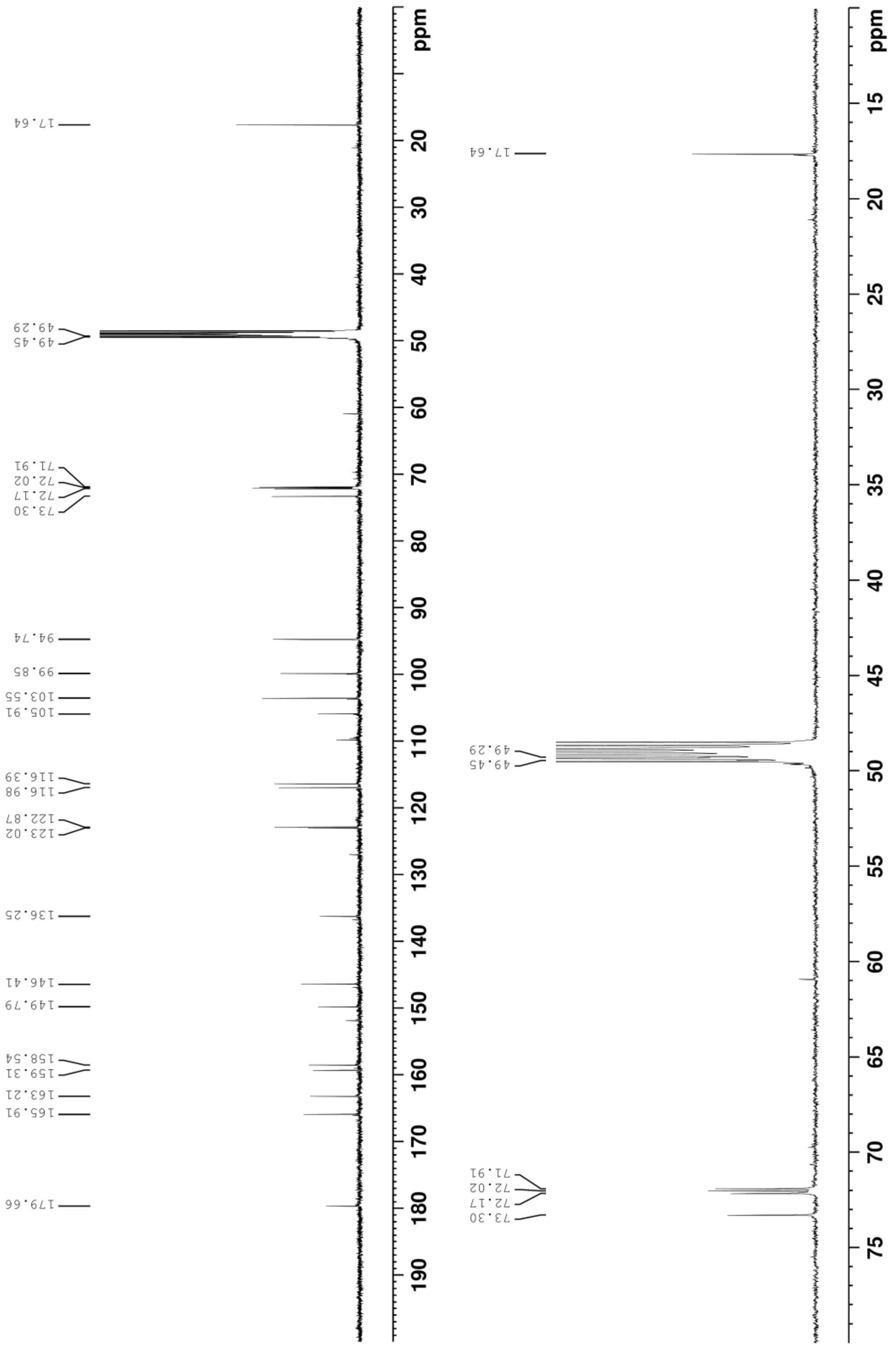

^{13}C-NMR spectrum of quercitrin (125 MHz, methanol-d_4)

The separation of the ethyl acetate extract of *S. antisepticum* leaves

1. The separation of EtOAc extract (17 g) to fractions A1-A6

Separation method: Short column chromatography with column diameter 12 cm

Stationary phase: Silica gel (Particle size 63–200 µm, 200 g)

Mobile phase: Hexane, 5% Acetone : Hexane, 10% Acetone : Hexane, 20% Acetone : Hexane, 30% Acetone : Hexane, 40% Acetone : Hexane, 50% Acetone : Hexane, Acetone, 5% MeOH : Acetone, 10% MeOH : Acetone, 15% MeOH : Acetone, 20% MeOH : Acetone, 30% MeOH : Acetone , 50% MeOH : Acetone, 70% MeOH : Acetone, and MeOH (1 L each)

2. The separation of fraction A4 (8.9 g) to fractions B1-B7

Separation method: Column chromatography with column diameter 7 cm

Stationary phase: Silica gel (Particle size 63–200 µm, 500 g)

Mobile phase: Hexane (1 L), 3% Acetone : Hexane (1 L), 5% Acetone : Hexane (1L), 10% Acetone : Hexane (2 L), 15% Acetone : Hexane (3.5 L), 20% Acetone : Hexane (5 L), 25% Acetone : Hexane (4 L), 30% Acetone : Hexane (2 L), 50% Acetone : Hexane (3 L), 70% Acetone : Hexane (1 L), Acetone (1 L), 30% MeOH : Acetone (1 L), 50% MeOH : Acetone (1 L), and MeOH (1 L)

3. The separation of fraction B4 (1.2 g) to fractions C1-C6

Separation method: Column chromatography with column diameter 5 cm

Stationary phase: Silica gel (Particle size 63–200 µm, 70 g)

Mobile phase: Hexane (200 mL), 5% Acetone : Hexane (200 mL), 10% Acetone : Hexane (400 mL), 15% Acetone : Hexane (400 mL), 20% Acetone : Hexane (400 mL), 25% Acetone : Hexane (200 mL), 30% Acetone : Hexane (100 mL), 50% Acetone : Hexane (100 mL), 70% Acetone : Hexane (100 mL), Acetone (100 mL), 30% MeOH : Acetone (200 mL), 50% MeOH : Acetone (100 mL), and MeOH (200 mL)

4. The separation of the mother liquor of fraction C3 (562.3 mg) to fractions D1-D7

Separation method: Column chromatography with column diameter 5 cm

Stationary phase: Silica gel (Particle size 63–200 µm, 70 g)

Mobile phase: Hexane (400 mL), 1% Acetone : Hexane (400 mL), 5% Acetone : Hexane (700 mL), 7% Acetone : Hexane (1.4 L), 10% Acetone : Hexane (1.2 L), 15% Acetone : Hexane (400 mL), 20% Acetone : Hexane (300 mL), 30% Acetone : Hexane (300 mL), 40% Acetone : Hexane (300 mL), 50% Acetone : Hexane (300 mL), 60% Acetone : Hexane (300 mL), 70% Acetone : Hexane (300 mL), 90% Acetone : Hexane (300 mL), Acetone (400 mL), 30% MeOH : Acetone (400 mL), 50% MeOH : Acetone (400 mL), 70% MeOH : Acetone (400 mL), and MeOH (500 mL)

5. The separation of fraction D3 (137.7 mg) to fractions E1-E7

Separation method: Column chromatography with column diameter 2.5 cm

Stationary phase: Silica gel (Particle size 63–200 µm, 30 g)

Mobile phase: Hexane (100 mL), 5% Acetone : Hexane (200 mL), 7% Acetone : Hexane (300 mL), 10% Acetone : Hexane (800 mL), 15% Acetone : Hexane (300 mL), 20% Acetone : Hexane, 30% Acetone : Hexane, 50% Acetone : Hexane, 70% Acetone : Hexane, 90% Acetone : Hexane, 100% Acetone, 50% MeOH : Acetone (200 mL each), and MeOH (400 mL)

6. The separation of fraction B6 (2.8 g) to fractions F1-F8

Separation method: Column chromatography with column diameter 5 cm

Stationary phase: Silica gel (Particle size 63–200 µm, 80 g)

Mobile phase: CH_2CL_2 (400 mL), 3% MeOH : CH_2CL_2 (750 mL), 5% MeOH : CH_2CL_2 (800 mL), 10% MeOH : CH_2CL_2 (500 mL), 15% MeOH : CH_2CL_2 (500 mL), 20% MeOH : CH_2CL_2 (500 mL), 25% MeOH : CH_2CL_2 (600 mL), 30% MeOH :

CH_2CL_2 (300 mL), 40% MeOH : CH_2CL_2 (300 mL), 50% MeOH : CH_2CL_2 (300 mL), 70% MeOH : CH_2CL_2 (300 mL), 90% MeOH : CH_2CL_2 (300 mL), and MeOH (800 mL)

7. The separation of fraction F3 (1.7 g) to fractions G1-G7

Separation method: Column chromatography with column diameter 3 cm

Stationary phase: Silica gel (Particle size 63–200 μm, 50 g)

Mobile phase: CH_2CL_2 (500 mL), 1% CH_2CL_2 : MeOH (400 mL), 3% CH_2CL_2 : MeOH (200 mL), 5% CH_2CL_2 : MeOH (200 mL), 10% CH_2CL_2 : MeOH (200 mL), 15% CH_2CL_2 : MeOH (200 mL), 20% CH_2CL_2 : MeOH (200 mL), 30% CH_2CL_2 : MeOH (200 mL), 40% CH_2CL_2 : MeOH (100 mL), 50% CH_2CL_2 : MeOH (100 mL), 60% CH_2CL_2 : MeOH (100 mL), 70% CH_2CL_2 : MeOH (100 mL), 90% CH_2CL_2 : MeOH (100 mL), and MeOH (500 mL)

8. The separation of fraction A5 (2.4 g) to fractions H1-H7

Separation method: Column chromatography with column diameter 3 cm

Stationary phase: Silica gel (Particle size 63–200 μm, 50 g)

Mobile phase: CH_2Cl_2 (1.4 L), 1% MeOH : CH_2Cl_2 (400 mL), 3% MeOH : CH_2Cl_2 (400 mL), 5% MeOH : CH_2Cl_2 (900 mL), 7% MeOH : CH_2Cl_2 (600 mL), 10% MeOH : CH_2Cl_2 (1.4 L), 20% MeOH : CH_2Cl_2 (500 mL), 30% MeOH : CH_2Cl_2 (500 mL), 40% MeOH : CH_2Cl_2 (400 mL), 50% MeOH : CH_2Cl_2 (400 mL), 60% MeOH : CH_2Cl_2 (400 mL), 70% MeOH : CH_2Cl_2 (400 mL), 80% MeOH : CH_2Cl_2 (300 mL), 90% MeOH : CH_2Cl_2 (300 mL), and MeOH (1.7 L)

9. The separation of fraction H5 (329.6 mg) to fractions I1-I5

Separation method: Column chromatography with column diameter 5 cm

Stationary phase: Silica gel (Particle size 40-63 μm, 50 g)

Mobile phase: CH_2Cl_2 (800 mL), 1% MeOH : CH_2Cl_2 (500 mL), 2% MeOH : CH_2Cl_2 (500 mL), 3% MeOH : CH_2Cl_2 (2000 mL), 4% MeOH : CH_2Cl_2 (1000 mL), 5% MeOH : CH_2Cl_2 (500 mL), 10% MeOH : CH_2Cl_2 (300 mL), 20% MeOH : CH_2Cl_2, 30% MeOH : CH_2Cl_2, 50% MeOH : CH_2Cl_2, 70% MeOH : CH_2Cl_2 ,90% MeOH : CH_2Cl_2 (200 mL each), and MeOH (500 mL)

The separation of the methanol extract of *S. antisepticum* leaves

1. The separation of MeOH extract (64.6 g) to fractions A1-A5

Separation method: Short column chromatography with column diameter 12 cm

Stationary phase: Silica gel (Particle size 63–200 μm, 400 g)

Mobile phase: CH_2Cl_2, 5% MeOH : CH_2Cl_2, 10% MeOH : CH_2Cl_2, 20% MeOH : CH_2Cl_2, 30% MeOH : CH_2Cl_2, 50% MeOH : CH_2Cl_2, 70% MeOH : CH_2Cl_2, and MeOH (2 L each)

2. The separation of fraction A4 (18.4 g) to fractions B1-B6

Separation method: Column chromatography with column diameter 10 cm

Stationary phase: Silica gel (Particle size 40-63 μm, 500 g)

Mobile phase: CH_2Cl_2 (1 L), 5% MeOH : CH_2Cl_2,10% MeOH : CH_2Cl_2, 20% MeOH : CH_2Cl_2, 30% MeOH : CH_2Cl_2, 40% MeOH : CH_2Cl_2, 50% MeOH : CH_2Cl_2, 55% MeOH : CH_2Cl_2, 60% MeOH : CH_2Cl_2, 65% MeOH : CH_2Cl_2, 70% MeOH : CH_2Cl_2, 75% MeOH : CH_2Cl_2, 80% MeOH : CH_2Cl_2, 85% MeOH : CH_2Cl_2, 90% MeOH : CH_2Cl_2 (800 mL each), and MeOH (2 L)

3. The separation of fraction B5 (3.6 g) to fractions C1-C9

Separation method: Column chromatography with column diameter 5 cm

Stationary phase: Silica gel (Particle size 40-63 μm, 70 g)

Mobile phase: CH_2Cl_2 (300 mL), 10% Acetone : CH_2Cl_2 (200 mL), 20% Acetone : CH_2Cl_2(200 mL), 30% Acetone : CH_2Cl_2 (200 mL), 40% Acetone : CH_2Cl_2 (200 mL), 50% Acetone : CH_2Cl_2 (200 mL), 60% Acetone : CH_2Cl_2 (200 mL), 65%

Acetone : CH_2Cl_2 (200 mL), 70% Acetone : CH_2Cl_2 (200 mL), 75% Acetone : CH_2Cl_2 (200 mL), 80% Acetone : CH_2Cl_2 2 (200 mL), 90% Acetone : CH_2Cl_2 (200 mL), Acetone (200 mL), 5% MeOH : Acetone (200 mL), 10%MeOH : Acetone (200 mL), 15%MeOH : Acetone (400 mL), 20% MeOH : Acetone (300 mL), 25% MeOH : Acetone (300mL), 30% MeOH : Acetone (300 mL), 40% MeOH : Acetone (300mL), 50% MeOH : Acetone (300mL), 60% MeOH : Acetone (300 mL), 70% MeOH : Acetone (300 mL), 80% MeOH : Acetone (300 mL), 90% MeOH : Acetone (300 mL), and MeOH (300 mL)

4. The separation of the precipitate obtained from fraction C3 (254.8 mg) to compound **6**

    Separation method: Column chromatography with column diameter 2.5 cm

    Stationary phase: Reverse phase silica gel C-18 (40 g)

    Mobile phase: H_2O : MeOH [1:1] (400 mL)

5. The separation of fraction C4 (613.7 mg) to fractions D1-D7

    Separation method: Column chromatography with column diameter 2.5 cm

    Stationary phase: Reverse phase silica gel C-18 (40 g)

    Mobile phase: H_2O : MeOH [1:1] (400 mL)

6. The separation of fraction D6 (83.6 mg) to fractions E1-E6

    Separation method: Column chromatography with column diameter 2.5 cm

    Stationary phase: Reverse phase silica gel C-18 (40 g)

    Mobile phase: H_2O : MeOH [1:1] (400 mL)
